# Assessing Intervention Effects in Sentence Processing: Object Relatives vs. Subject Control

**DOI:** 10.3389/fpsyg.2021.610909

**Published:** 2021-02-02

**Authors:** João Delgado, Ana Raposo, Ana Lúcia Santos

**Affiliations:** ^1^Research Center for Psychological Science, Faculdade de Psicologia, Universidade de Lisboa, Lisbon, Portugal; ^2^Centro de Linguística da Universidade de Lisboa, Departmento de Linguística Geral e Românica, Faculdade de Letras da Universidade de Lisboa, Lisbon, Portugal

**Keywords:** object relative clauses, subject control, intervention effects, generalized minimality, movement theory of control, individual differences, working memory, modularity

## Abstract

Object relative clauses are harder to process than subject relative clauses. Under Grillo’s (2009) Generalized Minimality framework, complexity effects of object relatives are construed as intervention effects, which result from an interaction between locality constraints on movement (Relativized Minimality) and the sentence processing system. Specifically, intervention of the subject DP in the movement dependency is expected to generate a minimality violation whenever processing limitations render the moved object underspecified, resulting in compromised comprehension. In the present study, assuming Generalized Minimality, we compared the processing of object relatives with the processing of subject control in ditransitives, which, like object relatives, instantiates a syntactic dependency across an intervening DP. This comparison is justified by the current debate on whether Control should be analyzed as movement: if control involves movement of the controller DP, as proposed by Hornstein (1999), a parallel between the processing of object relatives and subject control in ditransitives may be anticipated on the basis of intervention. In addition, we explored whether general cognitive factors contribute to complexity effects ascribed to movement across a DP. Sixty-nine adult speakers of European Portuguese read sentences and answered comprehension probes in a self-paced reading task with moving-window display, comprising four experimental conditions: *Subject Relatives*; *Object Relatives*; *Subject Control*; *Object Control*. Furthermore, participants performed four supplementary tasks, serving as measures of resistance to interference, lexical knowledge, working memory capacity and lexical access ability. The results from the reading task showed that whereas object relatives were harder to process than subject relatives, subject control was not harder to process than object control, arguing against recent movement accounts of control. Furthermore, we found that whereas object relative complexity effects assessed by response times to comprehension probes interacted with Reading Span, object relative complexity effects assessed by comprehension accuracy and reading times did not interact with any of the supplementary tasks. We discuss these results in light of Generalized Minimality and the hypothesis of modularity in syntactic processing (Caplan and Waters, 1999).

## Introduction

Comprehension of a relative clause requires resolution of a filler-gap dependency, i.e., establishing an interpretive dependency between an overt element of the sentence (filler) and an empty syntactic position in the sentence (gap). In subject relative clauses (SR, 1a), the nominal head (antecedent) of the relative clause (musician, in 1) is interpreted in the subject position of the embedded clause, whereas in object relative clauses (OR, 1b), it is interpreted in the direct object in the embedded clause.


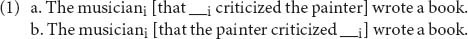


Interestingly, this structural difference is associated with an asymmetry in processing complexity, spanning different populations and linguistic contexts: comprehension of OR, but not of SR, is impaired in individuals with Agrammatic Aphasia (see Grodzinsky, 1984, 2000 and references therein) and OR are mastered later in development than SR (Friedmann et al., 2009 and references therein; Costa et al., 2011 for European Portuguese). In the case of adults, even for non-brain-damaged individuals, processing of OR is more costly than that of SR, as manifested in both online and offline measures (King and Just, 1991; Gordon et al., 2001, a.o.). The complexity asymmetry between SR and OR thus potentially qualifies as resulting from a universal parsing constraint (Kwon et al., 2013; Vasishth et al., 2013), rendering a unitary explanatory approach to OR complexity effects much desirable (Adelt et al., 2017).

In this study, assuming the main tenets of Generative Grammar, we adopt the Generalized Minimality (GM) framework proposed in Grillo (2005; 2008; 2009), which attributes the complexity effects of OR to the intervention of the subject determiner phrase (DP; the painter, in 1) in the movement relation^1^ established between the nominal head and the empty position. This will allow us to discuss whether similar effects should be and are effectively found in other structures in which we can identify intervention configurations. In the present paper, we will specifically compare intervention in OR structures and subject control across an intervening DP (subject control in ditransitive verbs). Considering this particular structure is made especially relevant by the ongoing debate in the Generative literature on whether control should be analyzed as movement (Hornstein, 1999).

In addition, we adopt an individual differences approach to explore whether general cognitive factors influence intervention effects in sentence processing, a question which remains largely unexplored. Possibly relevant cognitive factors include resistance to interference and lexical knowledge, under a cue-based parsing account of sentence processing (Lewis et al., 2006; Hofmeister and Vasishth, 2014), working memory capacity, under a capacity account of sentence processing (Gibson, 1998, 2000), and lexical access ability, assuming that the processing of structures in which there is intervention depends on timely access to morphosyntactic features (Grillo, 2009; Costa et al., 2012).

We therefore consider two general questions: (i) Do other structures involving filler-gap dependencies across an intervening DP induce complexity effects similar to those induced by OR? (ii) What are the cognitive factors underlying individual differences in complexity effects ascribed to movement across a DP?

### Generalized Minimality and the Processing Cost of Object Relatives

GM (Grillo, 2005, 2008, 2009) was initially formulated to account for comprehension deficits in Agrammatic Broca’s Aphasia, so called *canonicity effects*, i.e., selective difficulty in comprehending movement-derived sentences with non-canonical order of thematic role assignment (Caramazza and Zurif, 1976; Grodzinsky, 2000), e.g., OR, object wh-questions. Grillo (2005; 2008; 2009) treats canonicity effects as an interaction between locality constraints on movement and the sentence processing system. The right notion of locality here is that of Relativized Minimality (RM; Rizzi, 1990, 2004), which we now introduce.

According to RM, movement dependencies must be local, i.e., they must be satisfied in the minimal configuration in which they can be satisfied. Formally, we have that:


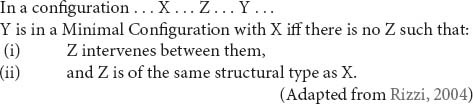


In this model, intervention is defined hierarchically: a constituent Z is said to intervene between X and Y if X c-commands Z and Z c-commands Y^2^. Structural type, on the other hand, has been defined in different ways. Here, we follow Grillo (2005; 2008; 2009) in adopting Rizzi’s (2004) approach in terms of specification of morphosyntactic features that define syntactic positions. Two elements are of the same structural type if their feature sets define the same type of syntactic position, i.e., belong to the same class^3^. See (3) for the typology of positions and their defining features assumed in Rizzi (2004) and Grillo (2005; 2008; 2009).


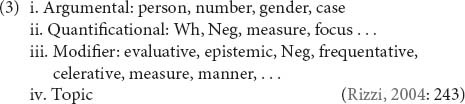


Thus, movement across a hierarchical intervener is predicted to be illicit whenever the moved element and the intervener belong to the same syntactic class. The application of this principle is illustrated by the wh-island in (4): movement of the question operator *how* across the intervening question operator *who* is blocked because both elements are specified with a wh-feature, therefore belonging to the quantificational class (see 3).


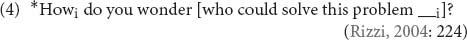


So, how can RM help understand canonicity effects? Consider the case of relative clauses (1, repeated in 5).


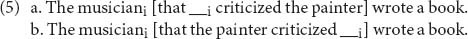


Assuming that movement targets the nominal head position (i.e., assuming a raising analysis of relative clauses, Kayne, 1994), OR instantiate a movement dependency which crosses an intervening element, the subject DP, whose feature set partially overlaps with that of the moved element, as both are specified with argumental features (e.g., phi-features). Importantly, as the nominal head is also specified with a *relative* feature (akin to the wh-feature in wh-questions), which defines the non-argumental position that it targets^4^, it does not share the structural type of the intervener, i.e., they do not belong to the same class (Grillo, 2008). Therefore, RM does not block the dependency. However, as Grillo (2005; 2008; 2009) points out, if the *relative* feature is not accessed during processing, OR will be represented as instantiating a movement dependency wherein the moved element and the intervening element are structurally identical, i.e., both belong to the argumental class. In such case, RM will block the dependency. Grillo thus proposes that the access to scope-discourse related features, such as those that motivate movement in relative clauses and wh-questions, is impaired in Agrammatic Aphasics, due to processing limitations. As a result of underspecification, canonicity effects emerge: structures involving movement across an intervening DP, such as OR, object wh-questions, object clefts and structures involving topicalization of objects, are atypically processed due to RM; on the other hand, structures in which the moved element does not cross an intervening DP, such as SR, subject wh-questions, subject clefts or unaccusative structures, are processed normally.

Importantly, GM can also apply to asymmetries in the comprehension of movement structures in children and healthy adults (Friedmann et al., 2009; see also Costa et al., 2012 and Belletti and Rizzi, 2013), therefore constituting a potential unitary account of OR complexity effects. According to GM, whenever the processing cost of accessing and maintaining activated the full array of morphosyntactic features distinguishing the moved element and an intervening DP is not payed, an intervention effect ensues, and comprehension suffers. From this perspective, larger OR complexity effects in Agrammatic Aphasics and children than in healthy adults result from weaker processing systems.

### Grammar and Processing of Obligatory Control Structures

As in the case of relative clauses, comprehension of an obligatory control structure^5^ requires resolution of a filler-gap dependency. In subject control with ditransitive verbs (SC, 6a), the matrix subject is interpreted as the filler (in more precise terms and in the case of control structures, the controller) of the gap, i.e., the empty embedded subject position, whereas in object control with ditransitive verbs (OC, 6b), the matrix object is interpreted as the controller of the empty subject position. Note that subject/object control reading is dependent on the matrix verb, i.e., the control verb: verbs like *promise* determine a subject control reading, whereas verbs like *convince* determine an object control reading.


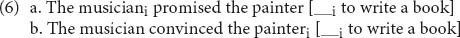


Despite being superficially similar to structures assumed to be derived via movement (e.g., subject and object raising, passives), control dependencies have received special treatment in Generative Grammar. This has been mainly motivated by the theta-criterion, which requires that the arguments in a sentence are in a one-to-one match configuration with theta-roles, therefore blocking movement from a theta-position to another. As control involves a dependency between two theta-positions, it has long been held that the empty subject position of a control structure is filled by a special type of null element, termed *PRO*, which is interpreted as anaphorically dependent on the controller (Chomsky, 1981). However, soon after the advent of the Minimalist Program, Hornstein (1999) argued for a movement theory of control (MTC), on the grounds of parsimony. In this framework, PRO is dispensed with, the theta-criterion is abandoned, and the work done by the theta-criterion is delegated to other components of the grammar.

It is beyond the scope of this article to assess the theoretical merits and shortcomings of movement and non-movement accounts of control (for discussion see Hornstein, 1999; Culicover and Jackendoff, 2001, 2006; Landau, 2003; Boeckx et al., 2010; Kirby et al., 2010). Instead, we consider predictions for processing which follow from adhering to the MTC, as opposed to more conservative theories, which postulate PRO. One such prediction is that SC, but not OC, may reveal processing complexity effects due to RM. If Control is derived through movement of the argument generated as an embedded subject to a position in the matrix clause (the matrix subject in the case of SC), as argued for by Hornstein (1999), this movement must be triggered by a relevant feature. In order to explain the possibility of SC in ditransitives, either there is no intervening DP or the intervening DP does not share a relevant feature. Hornstein and Polinsky (2010) and Boeckx et al. (2010) argue that there is no intervening DP. These authors assume that in English the benefactive argument of *promise* ([the painter] in 6a) is a PP headed by a null preposition. Since the DP would be embedded in the PP, it would not c-command the controlled subject position and therefore would not count as an intervener. If we do not accept this hypothesis and take the benefactive as a DP (we return to this later), this DP structurally qualifies as an intervener. In this case, in order to explain that subject control is possible in ditransitives, we must assume that the benefactive DP and the moved element do not share some relevant feature (exactly what we assume to explain object relatives). In this latter case, the prediction is that whenever underspecification of the type defined by Grillo is produced, we will find complexity effects in SC parallel to those found in OR.

Control structures have received considerably less attention inpsycholinguistic research than relative clauses. So, it is still not clear whether the structural difference between SC and OC is associated with a processing asymmetry. Studies with non-brain-damaged adults suggest a positive answer, even though the evidence is scarce^6^. Betancort et al. (2006) compared processing of SC and OC structures with ditransitive verbs in Spanish using eye-tracking (see (7) for a sample of the materials used^7^).


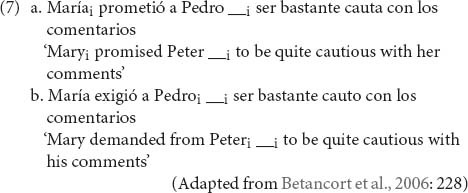


Results showed slower reading times for SC than OC at the complement preceding the empty position (i.e., *a Pedro*), both in early (first-pass reading times) and late reading measures (regression path times and total reading times). Assuming, as Betancort et al. (2006) suggest, that this difference may have reflected parafoveal processing of the subsequent region^8^ (containing the empty position), we can interpret these results as suggestive that: (i) The control dependency is resolved in the first region in which there is unambiguous evidence for the existence of an empty position; (ii) Resolution of the control dependency is more demanding for SC than for OC. However, even though there were no significant comprehension accuracy differences between conditions, the comprehension probes were presented for only one third of the trials and are not described in the paper, precluding conclusions about late stages of comprehension of control structures. More recently, Kwon and Sturt (2016) found that subjects have more difficulty reading sentences including giver control nominals than recipient control nominals. Although these data were obtained with control in nominal structures instead of control in complements of verbs, the converging results suggest that processing control dependencies across an intervening DP is costly. As in the study of Betancort et al. (2006), however, conclusions about later stages of comprehension of control structures are precluded by lack of information about the offline comprehension component of the task.

In addition, acquisition studies reveal a robust asymmetry, parallel to that of relative clauses, with SC mastered later in development than OC (e.g., Chomsky, 1969; Mateu Martin, 2016; Agostinho et al., 2018), which could justify the hypothesis that the same complexity constraints are operative in SC and OR. This comparison was directly explored by Martins et al. (2018), who compared the performance of typically developing children between 3 and 11 years of age, as well as two groups of atypically developing children between 8 and 11 years [children diagnosed with specific language impairment (SLI) and with autism spectrum disorder (ASD)] in two comprehension tasks testing subject and object relatives and subject and object control. The results in the two comprehension tasks confirmed that object relatives and subject control are, as expected, the problematic conditions in development. Nevertheless, different developmental patterns in relatives and in control structures were identified: the difficulties with object relatives are prolonged in childhood and still observable in the results of 8-11-year-olds; in contrast, there is an abrupt development of subject control, which causes difficulties to 5-7-year-olds, but not to 8-11-year-olds. Atypically developing children show diverging patterns with respect to the two problematic structures: ASD children showed more difficulty in comprehending subject control, whereas SLI children achieved lower comprehension results in the case of object relatives.

In sum, data on adult processing of control structures, though scarce, are indicative of a complexity asymmetry parallel to that of relative clauses, i.e., more difficulty in SC than in OC. Nevertheless, we stress that the scarcity of data from non-brain-damaged adult processing (especially the lack of data on offline measures of comprehension) significantly limits this conclusion. To the best of our knowledge, there is no previous work directly comparing processing of control structures and relative clauses and allowing to measure whether the same constraints are operative in subject control and object relatives. The acquisition results presented by Martins et al. (2018) are suggestive of different constraints on development.

### Processing Factors Underlying Complexity Effects in Movement-Derived Structures

We assume, following Grillo (2005; 2008; 2009), that OR complexity effects result from underspecification during processing, which justifies a RM effect. Identifying the processing factors that contribute to underspecification is therefore a question of interest. Grillo (2005; 2008; 2009) suggests that slower than normal activation of syntactic information may be one such factor in Aphasia, as evidenced by Agrammatic Aphasics’ slow lexical access and antecedent reactivation at movement trace positions (see Zurif et al., 1993 and references therein). Extending this hypothesis to non-brain-damaged adult processing, Costa et al. (2012) insist on the relevancy of timely access to a rich set of morphosyntactic features, associated to both the moved element and the intervener. In this case, one may expect that OR complexity effects are influenced by a subject’s lexical access ability.

As the surface configuration of OR, resulting from movement of the object, may be expected to impose memory difficulties, either due to capacity overload or interference, memory functioning might also qualify as a determinant of underspecification (Cilibrasi et al., 2019). According to Gibson’s (1998; 2000) Dependency Locality Theory (DLT), the memory cost of a dependency increases with its length, operationalized as the number of intervening new discourse referents^9^, which are held responsible for the bulk of processing cost in the construction of a sentence representation. This metric is justified by two assumptions, which follow from DLT’s adherence to a classical capacity-based view of working memory (Just and Carpenter, 1992; Baddeley, 2012): (i) the moved element is actively maintained in memory until the gap is found; (ii) there is a trade-off between maintenance and processing, i.e., both draw from, and thus compete for, the same pool of memory resources. Following this view, the larger the working memory capacity of the parser, the easier it should be to maintain the moved element of an OR across the intervening subject DP and verb, which introduce new discourse referents.

More recently, a growing number of researchers have shifted their attention from capacity-based explanations of memory costs in sentence processing to explanations based on similarity interference (Lewis et al., 2006; Van Dyke and Johns, 2012). This was mainly motivated by findings that the quality of memory representations affects sentence processing independently from quantity. Gordon et al. (2001), for instance, have shown that the subject/object asymmetry in relative clause processing is substantially reduced when the relative noun in the antecedent and the embedded noun (which is part of the direct object in SR and the subject in OR) mismatched in referential type – description vs. proper name or indexical pronoun (see Gordon et al., 2004 for similar results). These results have been attributed to similarity-based interference between the moved element and the intervening subject DP in cue-based parsing (Van Dyke and McElree, 2006; Hofmeister and Vasishth, 2014; Villata, 2017). According to this account, sentence constituents are not actively maintained in memory, but retrieved for integration via retrieval cues (for evidence that constituents are not actively maintained, see Nicol and Swinney, 1989; McElree et al., 2003; Foraker and McElree, 2011; but see also Wagers and Phillips, 2014; Ness and Meltzer-Asscher, 2017). Critically, retrieval is assumed to be content-addressable, such that the presence in memory of constituents similar to the target (i.e., sharing syntactic, semantic or referential features) is expected to compromise the retrieval process, either directly through retrieval interference (Van Dyke and McElree, 2006), or indirectly through encoding interference (Hofmeister and Vasishth, 2014; Villata, 2017). From this perspective, a subject’s capacity to resist interference may be expected to modulate complexity effects of OR (Tan et al., 2017). Lexical knowledge may also be expected to influence OR complexity effects in this view, assuming that subjects with greater lexical knowledge possess richer lexical representations, which are presumably less susceptible to interference (Van Dyke et al., 2014).

We now turn to the case of SC. As previously discussed, adopting the MTC, a parallel between OR and SC in terms of processing complexity can be anticipated, as underspecification of the moved element could justify a minimality effect during the processing of both structures. In this case, one may expect slow access to syntactic information to influence the processing of OR and SC in the same way. Furthermore, given the surface similarity between SC and OR, i.e., as in the dependency in OR, the dependency in SC crosses two new discourse referents, as defined in Gibson’s (2000) DLT (introduced by the matrix verb and the intervening object DP), and a constituent similar to the filler (intervening object DP), one would expect memory constraints, either based on capacity or interference, to equally contribute to underspecification in SC and OR, magnifying complexity effects to a similar extent in both structures. In sum, under Grillo; Grillo; Grillo’s (2005; 2008; 2009) model, the MTC leads to expect the same processing factors to equally influence the processing of SC and OR.^10^

So far, the discussion in the present section has been neutral as to the question of whether the system for syntactic processing is modular, i.e., functionally isolated (encapsulated), or dependent on domain-general mechanisms. However, predictions for interactions between syntactic processing effects and tasks assumed to measure more general cognitive factors, e.g., memory tasks, as advanced in the present study, are contingent on the position adopted in face of this question. Specifically, syntactic processing is expected to interact with general cognitive factors only insofar as the system responsible for such processing shares resources with other cognitive systems, i.e., is not modular. This has been mostly discussed in relation to working memory (but see James et al., 2018). In general, both evidence for and against modularity in syntactic processing has been documented (see Nicenboim et al., 2015; Tan et al., 2017; James et al., 2018; see also Adams and Gathercole, 2000 and Archibald, 2017, for a discussion pertaining to the role of working memory in acquisition), and the debate is far from settled. It is not possible to present a comprehensive review of the literature bearing on this question; rather, we focus on the research which most directly relates to the present work, i.e., studies involving relative clauses. Particularly relevant is the study by Caplan and Waters (1999), who reported several experiments in which no interaction between syntactic complexity effects (e.g., SR vs OR) and complex working memory span tasks (Daneman and Carpenter, 1980; Conway et al., 2005) was observed, leading them to propose that the memory system responsible for syntactic analysis and extraction of meaning from sentences is a cognitive module^11^ (for more recent results with relative clauses pointing in the same direction, see Caplan and Waters, 2005; Caplan et al., 2011; James et al., 2018; cf., King and Just, 1991). Following this view, effects of syntactic complexity (e.g., SR vs. OR) and working memory tasks are not expected to interact. The same reasoning may apply to other potential mechanisms deployed during syntactic processing, as might be the case of resistance to interference in memory (via, e.g., inhibition of memory competitors): adopting the view that syntactic processing is carried out by a cognitive module, it is not expected that general measures of resistance to interference interact with syntactic complexity effects.

### The Present Study: Aims and Hypotheses

The first aim of this study was to assess whether object relatives are paralleled by subject control in terms of processing complexity, as would be predicted by parallel analyses of relative clauses and control. To explore this question, we developed a sentence comprehension task, involving self-paced reading, in which participants read sentences and answered comprehension questions about the sentences they read. Complexity effects of syntactic processing were evaluated using three different measures: accuracy (i.e., proportion of correct responses to probe comprehension questions), response times (i.e., latency of response to probe comprehension questions), and reading times (i.e., time spent reading each word/region of the sentence). Assuming Generalized Minimality, we hypothesized that if control involves movement of the controller, parallel processing asymmetries between relative clauses and control should emerge as a result of intervention, i.e., more difficulty processing OR and SC than SR and OC, respectively.

The second aim of this study was to assess whether general cognitive factors modulate the processing of relative clauses and control structures. In addition to the reading task, participants performed four supplementary tasks: (i) the Brown-Peterson task (Kane and Engle, 2000), providing an index of resistance to interference, more specifically, resistance to proactive interference (Friedman and Miyake, 2004; Pettigrew and Martin, 2014), which resembles interference in sentence processing in that it requires inhibition of memory competitors (Tan et al., 2017); (ii) the vocabulary subtest of the WAIS-III (Wechsler, 2008), as a proxy of lexical knowledge, which, in turn, was expected to relate positively with the richness of subjects’ lexical representations (Van Dyke et al., 2014); (iii) the Reading Span (Kane et al., 2004), providing an index of working memory capacity; (iv) and the semantic fluency task (Troyer et al., 1997), as a measure of lexical access ability. We hypothesized that if the processes involved in intervention effects are not modular (i.e., encapsulated), complexity effects of object relatives should interact with supplementary tasks.

An interaction between the fluency task and OR complexity effects is expected if underspecification results from slow access to syntactic information. Furthermore, an interaction between Reading Span and OR complexity effects is expected if underspecification results from memory overload. Interactions between the Brown-Peterson task and lexical knowledge and OR complexity effects are predicted if underspecification results from interference in memory. If, however, Caplan and Waters (1999) modularity hypothesis is held, no interactions between memory tasks and OR complexity effects are expected.

Finally, we aimed at determining whether the same cognitive factors underly the processing of OR and SC. We therefore hypothesized that if relatives and control both involve syntactic movement, the same cognitive factors that may be found to modulate the processing cost of object relatives would also modulate the processing cost of subject control.

## Methods

### Participants

Seventy-four (68 females) healthy participants took part in the study. All were right-handed, native speakers of European Portuguese, with no language or reading disability, and had normal or corrected to normal vision. Participants’ ages ranged from 17 to 41 years old (*M* = 19.7, *SD* = 4.4). All gave informed consent to the experimental procedure, which was approved by the Ethics Committee of the Faculty of Psychology of the University of Lisbon.

Five participants were excluded from the analysis due to atypical performance in the sentence comprehension task: two participants had low accuracy in answering sentence comprehension probes (lower than 67%), one participant had exceptionally long response times (with mean response time greater than 3 standard deviations of the sample mean), one participant had exceptionally long reading times (with mean reading time greater than 3 standard deviations of sample mean) and one participant reported failing to read naturally (by consciously trying to memorize every sentence). The remaining sixty-nine participants constituted the final sample.

### Materials and Procedure

The tasks were administered individually in a single session, lasting approximately 1.5 h. Testing was conducted in a quiet room and computerized tasks were always run in the same computer. Participants were told that they could take intervals between the tasks whenever necessary. The order of the tasks was kept constant for all participants, to avoid confounding individual differences with task order effects: Sentence comprehension task, Brown-Peterson task, vocabulary subtest (WAIS-III), Reading Span, semantic fluency task.

#### Sentence Comprehension Task

##### Materials

We constructed 30 pairs of sentences with relative clauses, one a subject relative clause [SR condition, see (8a)] and the other an object relative clause [OR condition, see (8b)]. Another 30 pairs of sentences with control dependencies were also created, one a subject control dependency [SC condition, see (9a)] and the other an object control dependency [OC condition, see (9b)]. We present a simplified representation of the sentences in each condition. For a complete list of the experimental sentences, see Appendix I in the Supplementary Material.


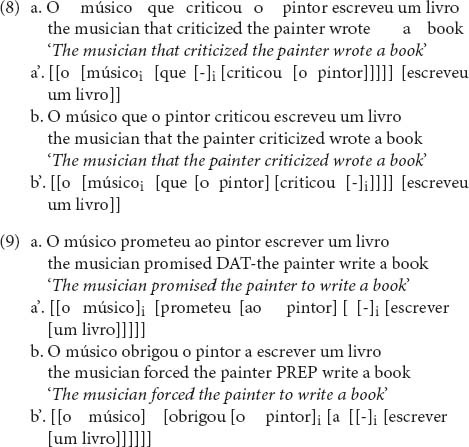


Superficially, sentences with relative clauses within each pair differed only in the order of the words inside the relative clause: the embedded verb could either precede the DP, yielding a sentence with a SR, or follow the DP, yielding a sentence with an OR.^12^ All sentences in SR and OR conditions were 9 words long and had the same structure as those in (8). Sentences with control dependencies within each pair differed in the type of control verb (i.e., main verb): whereas sentences in the SC condition were constructed with subject control ditransitive verbs, sentences in the OC condition were constructed with object control verbs. Since there is a very limited number of ditransitive verbs that are subject control verbs in European Portuguese, only five control verbs could be used for constructing the thirty SC sentences (*jurar* “swear,” *prometer* “promise,” *assegurar* “assure,” *ameaçar* “threaten,” *garantir* “guarantee”) and another five control verbs were used for constructing the thirty OC sentences (*obrigar* “obligate”“force,” *forçar* “force,” *convencer* “convince,” *autorizar* “authorize,” *encorajar* “encourage”). SC sentences with *ameaçar*, such as (10), as well as OC sentences (see 9b) were 9 words long, whereas the remaining sentences with control dependencies were 8 words long. This difference was due to the occurrence of an extra preposition introducing the embedded clause in OC sentences and in SC sentences with *ameaçar*.


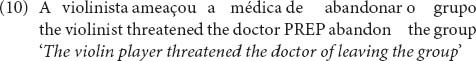


A difference between SC and OC sentences should be highlighted: the indirect object in SC structures is introduced by the element *a*, homophonous with the preposition *a.* In this case, if we analyze this *a* as a preposition, in line with the null preposition analysis of English benefactives suggested by Hornstein and Polinsky (2010) and Boeckx et al. (2010), no DP intervenes between the controller and the empty position (see the discussion centered on English in Section “Grammar and Processing of Obligatory Control Structures”). We assume instead that *a* is a Dative case marker, along the lines of Vergnaud (1974); Larson (1988) for English dative *to* and Gonçalves (2015), specifically for Portuguese *a.* Therefore, the benefactive [ao pintor] in (9a) counts as a DP which c-commands the embedded subject position. The argument structure of the subject control verb *ameaçar*, “threaten,” is again an exception: its DP argument bears Accusative case, and is therefore not marked by *a.* Apart from Dative marking and the difference in control verbs, pairs of SC and OC sentences were identical (considering the noun phrases used, as well as the embedded verb), and had the same structure as those in (9).

Wherever possible, we used the same lexical material for constructing sentences in relative clause conditions and sentences in control conditions, as illustrated in (8) and (9). Sentences were thus constructed in sets, such that each set contained a pair of sentences with relative clauses and a pair of sentences with control dependencies. Sentences from the same set contained the same DPs. The embedded verbs differed, since forcing sentences with relative clauses and sentences with control dependencies to have the same embedded verbs would compromise plausibility. Importantly, embedded verbs in sentences with relative clauses and sentences with control dependencies were matched in number of syllables, *t*(58) = −1.027, *p* = 0.309, and number of characters, *t*(58) = −0.623, *p* = 0.535. The main verbs also differed. The main verbs of sentences with relative clauses were the verbs used in the complement clauses of control sentences, to maximize content identity across sentences with relative clauses and control sentences, whereas the main verbs of control sentences were necessarily control verbs. NP1 (i.e., *músico*) and NP2 (i.e., *pintor*) were always descriptions of professions matched in gender and number, which were selected so that there were no inherent authority relationships between them, which could bias interpretation of control structures (e.g., with verbs such as *force*: for instance, it is plausible that a judge would force a lawyer to do something, but not that a lawyer would force a judge to do something). The verbs (except for control verbs) were always common transitive verbs describing actions. NP3 (i.e., *livro*) was always a description of an inanimate entity. All experimental sentences were constructed so that there were no inherent semantic relationships between NP1 and NP2 and the verbs (e.g., if the profession *writer* were included in a sentence, propositions related to writing would be avoided).

To reduce potential exposure effects, participants saw only two sentences from each set, one with a relative clause and one with a control dependency. In total, participants saw 15 sentences from each condition (SR, OR, SC, and OC). In sentences with control dependencies, participants saw each control verb three times. Sixty additional sentences were included as fillers (structures involving coordination, finite complement clauses and temporal clauses). Fillers were syntactically different from the experimental sentences but had equal number of clauses and similar length. Filler sentences were included to divert participants’ attention from the manipulations of interest and reduce the likelihood of adoption of conscious strategies in parsing.

##### Procedure

Sentences were presented in a word-by-word self-paced manner, with a moving window display. The experiment was constructed and run using *PsychoPy* software (Peirce et al., 2019). All sentences were presented individually and occupied a single line on the center of the screen. Each trial started with a fixation cross presented at the center of the screen for 500 ms, followed by a series of underscores corresponding to the number and length of words in the sentence. Participants pressed the space bar to reveal each hidden word. When each word was revealed, the previous word reverted to underscores. Reading times at each word were measured as the time between key presses. Participants were asked to read attentively and at a natural pace. After reading each sentence, participants were shown a comprehension probe statement about the content of the sentence they had just read and were asked to press a *yes* key (signaled in green) if the statement was true and a *no* key (signaled in red) if the statement was false, as accurately and fast as possible. Accuracy and response times were also recorded. Comprehension probes were clefts that always targeted the critical dependencies in experimental sentences, i.e., resolution of the dependency between the antecedent (filler) and the empty position (gap). Clefts like (11) were used for sentences with relative clauses and clefts like (12) were used for sentences with control dependencies.


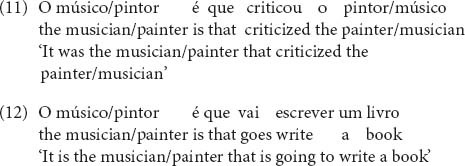


We used clefts instead of simpler sentences in order to avoid a potential problem with control conditions: most control sentences that we used do not provide sufficient information for the reader to know whether the entity corresponding to the controlled subject has performed/will perform the action described by the embedded verb. For instance, the sentence *The musician promised the painter to write a book* does not entail that the musician will actually write a book – he may have lied. Therefore, participants could have answered false to a true probe statement such as *The musician is going to write a book.* Cleft probes, such as *It is the musician that is going to write a book*, allow us to circumvent this problem by putting a DP in focus (i.e., *the musician*) and presupposing that the event described by the rest of the sentence is true (i.e., *someone is going to write a book*), therefore prompting participants to decide which DP functions as the subject of the embedded clause, which is precisely what we aimed to assess. Since only subject clefts (not object clefts) were used to test accuracy, no intervention effects were expected in the comprehension of the cleft probe itself. Finally, we used *é que* clefts due to their frequency in spontaneous speech, especially when a subject is clefted (Lobo et al., 2016).

For each sentence, a true and a false probe statement were constructed to control for potential response bias. Each participant saw only one comprehension probe per sentence and all participants saw an equal number of true and false probes. Before the experiment began, participants performed four practice trials to get familiar with the task. Following the practice trials, participants saw four blocks of 30 sentences, each with a similar number of sentences from each experimental condition. Within-block sentence presentation order was randomized for each participant and block presentation order was counterbalanced.

#### Supplementary Tasks

##### Brown-Peterson task

The Brown-Peterson task measures resistance to proactive interference (PI). The task comprised three blocks. In each block, participants were presented with four lists of eight words. The first three lists consisted of exemplars taken from the same semantic category (mammals, countries, or body parts), building up interference from List 1 to List 3 (i.e., as the number of similar items in memory increases from List 1 to List 3, it is gradually more difficult to retrieve the items from a specific list, due to interference from previous lists), whereas the fourth list consisted of exemplars taken from a different category (clothes, fruits, or types of dwellings), allowing a release from interference. The words used in the lists were taken from the Portuguese category norms in Pinto (1992). Words in each category were ranked below the 12 strongest associates to the category and had at most 10 letters, following Kane and Engle (2000).

The task began with a fixation cross displayed on the center of the screen for 2 s, followed by a list of eight words, presented word-by-word on the center of the screen at the rate of 2 s (1,750 ms for stimulus presentation + 250 for interstimulus interval). Participants read each word aloud. After seeing a complete list, participants performed a distractor task: a letter-number pair (e.g., B-20) typed in blue appeared on the center of the screen and participants immediately read aloud the letter-number pair and named the subsequent pairs according to the alpha-numeric sequence, as fast and accurately as possible (e.g., if presented with the pair B-20, participants should say: B-20, C-21, D-22, E-23, …). After 16 s, a black screen with two question marks (*??*) cued participants to orally recall the words from the list they saw. They had 20 s to recall as many words as they could, in any order. When the recall period was over, a screen with the phrase *Terminou o tempo* “The time is up” appeared for 2 s, after which the sequence described above was repeated, starting with the fixation cross (indicating an upcoming list of words). Participants had 15 s to rest between blocks.

Block and interference build-up list presentation order were randomized for each participant. Presentation and timing of stimuli were controlled using *E-Prime* software^13^. The dependent measure was the mean proportional interference effect, calculated for each participant by subtracting the mean recall on List 3 from the mean recall on List 1 and dividing the remainder by the mean recall on List 1 (similarly to Kane and Engle, 2000). Higher scores indicate increased reduction in correct recall relative to List 1 (the baseline, wherein there is no interference), and thus a lower capacity to resist interference.

##### Vocabulary subtest (WAIS-III)

We used the vocabulary subtest of the WAIS-III (Wechsler, 2008) as a measure of verbal knowledge. Participants were asked to provide definitions for up to 33 words. We followed the administration and scoring criteria provided in the WAIS-III manual. Each answer received a score of 0, 1, or 2, where *0* indicates a clearly incorrect answer, *1* indicates an answer which, though not incorrect, reveals poverty in content, and *2* indicates an answer that reflects good understanding of the meaning of the word. The dependent measure was the total score in the test, summed across the 33 words, with higher scores denoting more extensive verbal knowledge.

##### Reading span

The Reading Span task was used to obtain an index of working memory capacity. Participants were required to remember letters while performing a reading task, based on Kane et al. (2004). Participants were shown Power-point slides containing a single sentence followed by a question mark and a to-be-remembered letter (e.g., *A camisola branca fica-lhe larga, mas a preta fica-lhe provável* “The white sweater is loose on him, but the black one is probable” *? X*), centered onscreen. Each sentence consisted of 10–15 words, was unrelated to the others, and could be either understandable or nonsensical. Nonsensical sentences were rendered nonsensical by a semantically or pragmatically incongruent word (e.g., *probable* in the example above), which could appear equally often in the beginning, middle and end of the sentence. There was an equal number of understandable and nonsensical sentences. The letters used were *B*, *L*, *J*, *F*, *X*, *Q*, *M*, *R*, *H*, following Kane et al. (2004). These letters were chosen because their names are phonologically distinct from each other. Each letter appeared an equal number of times in the experiment and no more than once in each trial.

When participants were shown a slide containing a sentence, they immediately started reading aloud. After reading the sentence, they verified aloud whether it made sense, by saying *yes* if it made sense and *no* if it did not. The question mark was included in the display to remind participants to give their answer. Finally, they read aloud the letter and the experimenter switched slides. Each trial consisted of a set of two to five sentence-letter sequences. After seeing all the sentence-letter sequences in a trial, participants saw a recall cue (black screen with two question marks), indicating that they should write all the letters seen in the current trial in a response sheet, in the order that they appeared.

There were three trials for each set size (2–5), for a total of twelve trials. Trial presentation order was randomized once. The dependent measure was the mean proportion of correctly recalled elements (letter and position) per trial (Conway et al., 2005). Higher scores reflect greater working memory capacity.

##### Semantic fluency task

To measure lexical access ability, we used the semantic fluency task. Participants were given a semantic category (e.g., supermarket items) and were asked to name as many exemplars of that category as possible in 60 s. There were two semantic categories, *supermarket items* and *vegetables*. We chose these categories because they are broadly used in the literature (e.g., Clark et al., 2014) and do not overlap with the categories used in the Brown-Peterson task. The dependent measure was the mean number of productions, excluding errors and repetitions (Troyer et al., 1997). When a subcategory (e.g., fish) was produced along with specific members of that category (e.g., salmon, swordfish, monkfish), only the specific exemplars (salmon, swordfish, monkfish) were counted, following Troyer and Moscovitch (2006).

### Data Analysis

The analyses of response time and reading times were restricted to correct trials. Response times that were more than 3 standard deviations away from the mean of each condition, by participant, were excluded (affecting 0.4% of the data). Reading times that were more than 3 standard deviations away from the mean of each word position, by condition and participant, were removed, and multi-word regions including removed words were excluded from the analysis (affecting 0.7–2.2% of the data, depending on region). Response times and reading times were log-transformed to improve the normality of the residuals (so as to conform with the assumptions of the general linear model, Baayen and Milin, 2010).

For the analysis of reading times in relative clause conditions, we defined two regions of interest:

1. The *critical region* was defined as the last three words of the relative clause (the complementizer was omitted because it is the same in SR and OR, following previous studies), which contains the same words in SR and OR, only in a different order, thus allowing for a straightforward contrast between subject- and object-gap filling.

2. The *post-critical region* was defined as the two words that follow the relative clause region, the main verb and the determiner from the last DP, in which there may still be processing related to the resolution of the filler-gap dependency (i.e., spillover effect, Just et al., 1982). The noun from the last DP was not included because reading times at the last word may reflect processes related to revision of the whole sentence (i.e., *wrap-up effects*, Just and Carpenter, 1980).

For the analysis of reading times in control conditions, we defined one region of interest:

1. The *critical region* was defined by discarding the last word of the sentence, for the reason pointed out before, and selecting the last two words in SC (Embedded verb + Determiner from DP3) and the last three words in OC (preposition *a* + embedded verb + determiner from the last DP). Although the empty position is assumed to occur adjacent to the embedded verb position in both SC and OC sentences, a direct comparison between these two words would suffer from interpretability issues. In OC sentences, the preposition that occurs before the embedded verb (which is absent in SC sentences, except for those with *ameaçar*) unambiguously informs the parser that an empty position is coming next: from the preposition, the embedded verb necessarily follows. In SC sentences, on the other hand, the parser does not know where the empty position is until reaching the embedded verb position (since the noun that precedes the embedded verb is modifiable). It is reasonable to assume, then, that assignment of a DP as controller of the empty position may initiate at the preposition in OC (the first region of direct evidence for the empty position) and at the embedded verb in SC (this hypothesis is consistent with the reading time data presented in Figure 7, showing that whereas the reading time peak for SC sentences occurs at the embedded verb, the reading time peak for OC sentences occurs at the preposition preceding the embedded verb). If this reasoning is on the right track, then reading times at the embedded verb region may reflect different processes in SC and OC sentences. We therefore took the whole complement clause (excluding the last noun, due to potential wrap-up effects, as previously mentioned), including the preposition in OC, as the critical region, containing all positions in which there may be processing related to resolution of the control dependency (including post-verbal regions, wherein there may be spillover effects from the preceding regions – embedded verb in SC and preposition + embedded verb in OC – Just et al., 1982). Although we argue that this contrast is more justified than a direct comparison between embedded verbs, the extra word in OC sentences (i.e., preposition) still renders interpretation complicated (even though the dependent measure was, as noted before, the average reading time at each region of interest). We return to this point in the discussion.

The data were analyzed using Mixed effects models (Locker et al., 2007; Baayen et al., 2008), constructed in SPSS 25. For the analysis of accuracy, a logit link function was used (Jaeger, 2008). The models for accuracy and response times aimed to assess whether the predicted difference between SR and OR is paralleled in control structures. These models included all the syntactic conditions (i.e., SR, OR, SC, and OC), comprising as fixed effects the main effects of the syntactic variables *Structure type* (*Control*; *Relative clause*) and *Structure subtype* (*Subject*; *Object*) and their interaction. In the analysis of accuracy, an additional variable, i.e., *Expected answer* to the comprehension probe (*True*; *False*), was also included, so as to examine response bias in our sample, since it may interfere in the estimation of the effects of syntactic variables. Both the main effect of this variable and all possible interactions with the syntactic variables were entered as fixed effects. Reading times were analyzed with separate models for relative clause and control conditions, as the sentence structures necessarily differed. These models tested for subject/object asymmetries in online resolution of the filler-gap dependencies in relative clauses and control, including as fixed effect the main effect of the variable *Structure subtype* (*Subject*; *Object*).

Follow-up models were constructed to assess whether significant contrasts between relative clause conditions (SR vs OR) or control conditions (SC vs OC) were modulated by supplementary tasks. In addition to the main effect of *Structure subtype* (*Subject*; *Object*), these models included as fixed effects the main effects of individual differences in all supplementary tasks and all possible interactions between individual differences and *Structure subtype*. Since all supplementary tasks were always included in the models, the models tested for the effect of each task while controlling for the effects of the other tasks (i.e., unique effect). Three participants were excluded from these analyses, as they had missing data on the semantic fluency task due to an error in the recording of their responses. Full results of the follow-up models are reported in Appendix II of the Supplementary Material.

Participants and items were entered as random effects in the models wherever possible. Categorical variables were effect coded by SPSS. Continuous independent variables (individual differences in supplementary tasks) were mean centered prior to analysis. Hence, significant effects of syntactic variables in models that include individual differences in supplementary tasks indicate effects of syntax when performance in the supplementary tasks is at the mean.

To assess whether the data from the supplementary tasks were consistent with the previous literature, we computed Bivariate Pearson correlations between the tasks. This also allowed us to probe for potential multicollinearity between our tasks. In addition, we explored whether the performance with the Brown-Peterson task at the group level replicated previous research, thus justifying the assumption that this task is indeed sensitive to similarity interference. We begin the following section (Results) by presenting these analyses, together with descriptive statistics of the supplementary tasks. Then, we report, in turn, the results obtained in the mixed effects models for accuracy, response time and reading times.

## Results

### Supplementary Tasks

**FIGURE 1:**
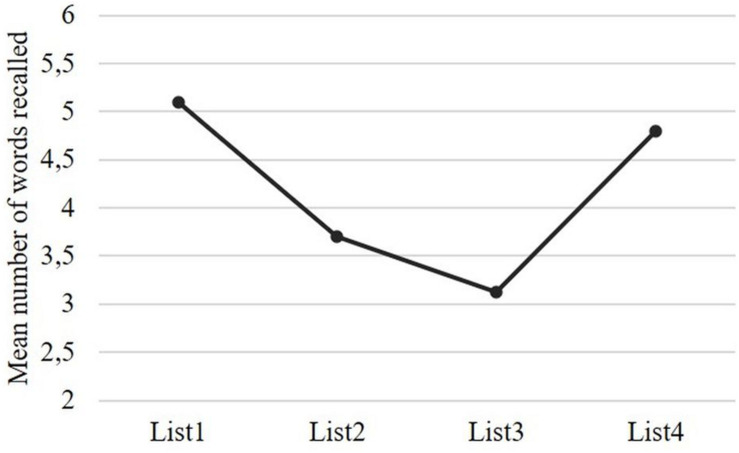
Mean number of words recalled per list in the Brown-Peterson task.

Figure 1 shows the mean performance on the Brown-Peterson task. Performance on the Brown-Peterson task decreased from List 1 to List 2 and from List 2 to List 3, evidencing interference buildup, and increased from List 3 to List 4, evidencing release from interference. This replicates previous research (e.g., Kane and Engle, 2000; Friedman and Miyake, 2004) and indicates that performance on the Brown-Peterson task was sensitive to similarity-interference, as predicted.

Table 1 presents the overall mean scores, standard deviations and range found in all supplementary tasks.^14^ Table 2 presents bivariate Pearson correlations between the measures.

**TABLE 1:** Descriptive statistics for the supplementary tasks.

Task	Mean	SD	Range	Max. score
Brown-Peterson task	0.38	0.19	*−*0.22 to *−*0.85	1
Vocabulary subtest	47.03	6.32	34 to 60	66
Reading Span	0.62	0.13	0.25 to *−*0.95	1
Semantic fluency task	13.13	4.19	5 to 28.5	–

**TABLE 2:** Bivariate Pearson correlations between the supplementary tasks.

Task	Brown-Peterson task	Vocabulary subtest	Reading Span	Semantic fluency task
Brown-Peterson task	–	–	–	–
Vocabulary subtest	−0.117	–	–	–
Reading Span	−0.259*	0.202	–	–
Semantic fluency task	−0.346**	0.290*	0.160	–

*Significant at the 0.05 level; **Significant at the 0.01 level.

The Brown-Peterson task showed a weak, yet significant negative correlation with Reading Span (*r* = −0.259, *p* < 0.05), consistent with prior work and theoretical models that postulate an attention component in working memory capacity which guards processing against interference (Engle and Kane, 2004; Borella et al., 2006; Unsworth, 2010). The semantic fluency task was significantly correlated with both the vocabulary subtest (*r* = 0.290, *p* < 0.05) and the Brown-Peterson task (*r* = −0.346, *p* < 0.01), suggesting that semantic fluency is associated with lexical knowledge as well as with the ability to resist interference (Rosen and Engle, 1997). Since all correlations were modest (strongest correlation: *r* = −0.346), there was no evidence for multicollinearity between our measures.

### Comprehension Accuracy

**FIGURE 2:**
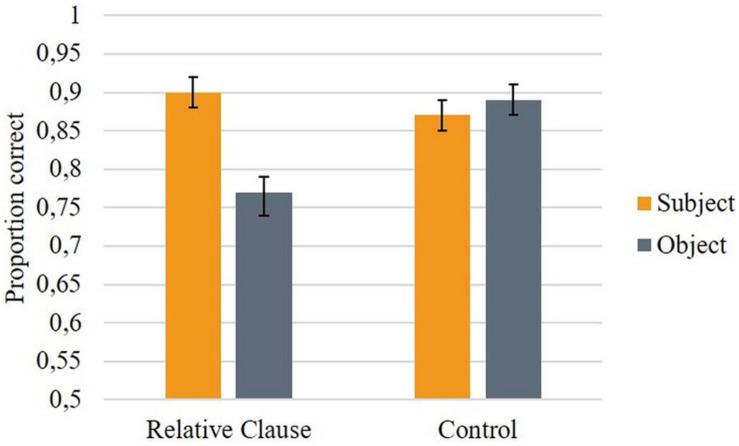
Mean proportion of correct responses per condition (bars represent 95% confidence interval).

Participants’ overall accuracy was high (*M* = 88%, *SD* = 7.7%, across experimental items and fillers), indicating that they performed the task attentively. Figure 2 shows the mean proportion of correct answers per condition.

Accuracy was not significantly different as a function of the expected answer to the comprehension probe, *F*(1, 4132) = 3.46, *p* = 0.063. Importantly, the triple interaction between *Structure type*, *Structure subtype* and *Expected answer* to the comprehension probe was also not significant, *F*(1, 4132) = 0.026, *p* = 0.871. These results suggest that response bias was negligible and did not interfere with the syntactic variables. Therefore, we did not include the effect of *Expected Answer* to the comprehension probe in any other analysis. The critical interaction between *Structure type* and *Structure subtype* was significant, *F*(1, 4132) = 29.72, *p* < 0.001. Pairwise Sidak-corrected contrasts showed that whereas accuracy for SR was significantly greater than for OR, *t*(4132) = 6.024, *p* < 0.001, there was no difference between SC and OC, *t*(4132) = −1.336, *p* = 0.182. A follow-up model for relative clauses assessed whether the effect of *Structure subtype* was modulated by supplementary tasks. There were no significant interactions.

**FIGURE 3:**
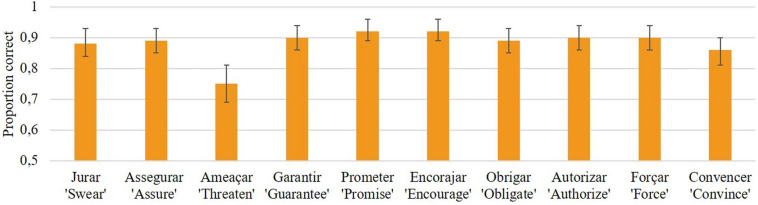
Mean proportion of correct responses by control verb (bars represent 95% confidence interval).

Since the properties of the control verbs were not controlled for (e.g., frequency), due to the limited number of subject control verbs in European Portuguese, we conducted two *post hoc* analyses, one only with SC sentences and another only with OC sentences, to assess whether accuracy changed as a function of the control verb used in the sentence – it could be that some verbs were more representative of their category than others. Results for the SC condition^15^ revealed a significant main effect of control verb, *F*(4, 1030) = 7.61, *p* < 0.001. Pairwise Sidak-corrected comparisons indicated that the SC verb *ameaçar* “threaten” differed from all other subject control verbs (see Table 3 and Figure 3). No other comparison emerged as statistically significant. Results for the OC condition revealed no significant main effect of control verb, *F*(4, 1030) = 1.18, *p* = 0.319. These findings suggest that control structures with *ameaçar* were processed atypically. Therefore, we excluded all such sentences from the remaining analyses.

**TABLE 3:** Pairwise Sidak-corrected contrasts for subject control verbs.

Contrast	t-statistic	*df*	*p*-value
Jurar – Assegurar	−0.16	1030	0.951
Jurar – Ameaçar	3.48	1030	0.004
Jurar – Garantir	−0.48	1030	0.951
Jurar – Prometer	−1.34	1030	0.701
Assegurar – Ameaçar	3.63	1030	0.002
Assegurar – Garantir	−0.32	1030	0.951
Assegurar – Prometer	−1.18	1030	0.743
Ameaçar – Garantir	−3.93	1030	0.001
Ameaçar – Prometer	−4.71	1030	<0.001
Garantir – Prometer	−0.86	1030	0.859

### Response Time

**FIGURE 4:**
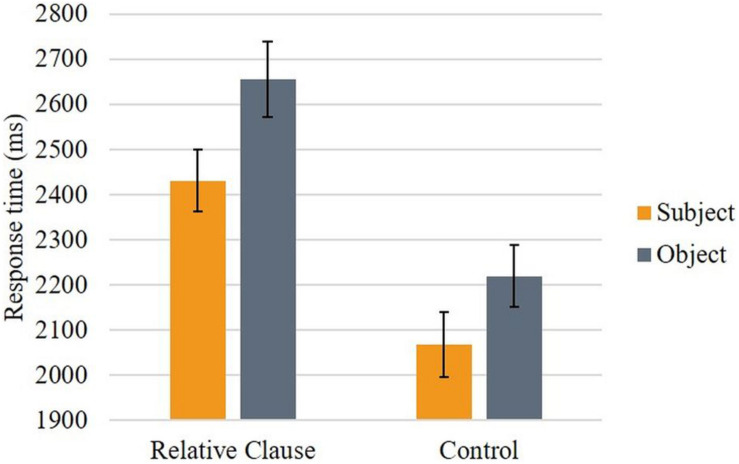
Mean response time by condition (bars represent 95% confidence interval).

Figure 4 shows the mean response time to the comprehension probe per condition.

**FIGURE 5:**
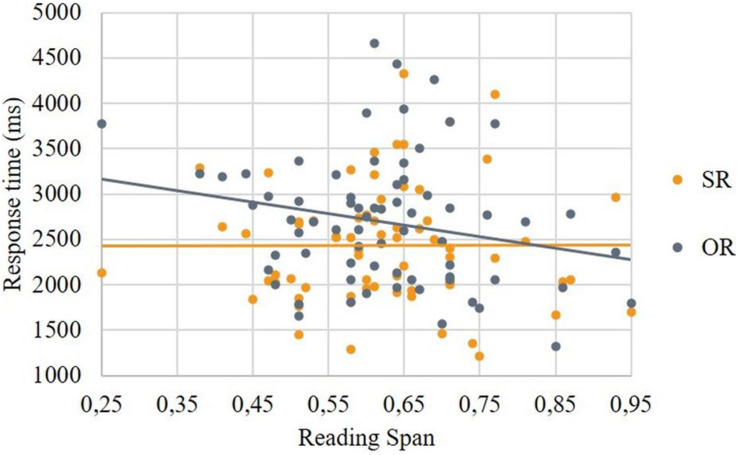
Correlation between Reading Span and response time in relative clause conditions.

The results showed that the interaction between *Structure type* and *Structure subtype* was not significant, *F*(1, 3379) = 0.082, *p* = 0.774. Pairwise Sidak-corrected contrasts showed that the difference in response time between SR and OR was significant, *t*(3379) = −3.46, *p* < 0.001, with longer response times for OR, and that the difference in response time between SC and OC was also significant, *t*(3379) = −2.94, *p* = 0.003, with longer response times for OC. The non-significant interaction indicates that these differences did not differ. Follow-up models for relative clause conditions and control conditions assessed whether the effects of *Structure subtype* were modulated by supplementary tasks. Whereas the model for control conditions did not reveal a significant interaction, the model for relative clauses revealed a significant interaction between *Structure subtype* and Reading Span, *F*(1, 1645) = 5.37, *p* = 0.021. As illustrated in Figure 5, this interaction reflected a stronger impact of Reading Span on response times for OR than for SR sentences, with higher Reading Span scores associated with shorter response times for OR.

### Reading Times

**FIGURE 6:**
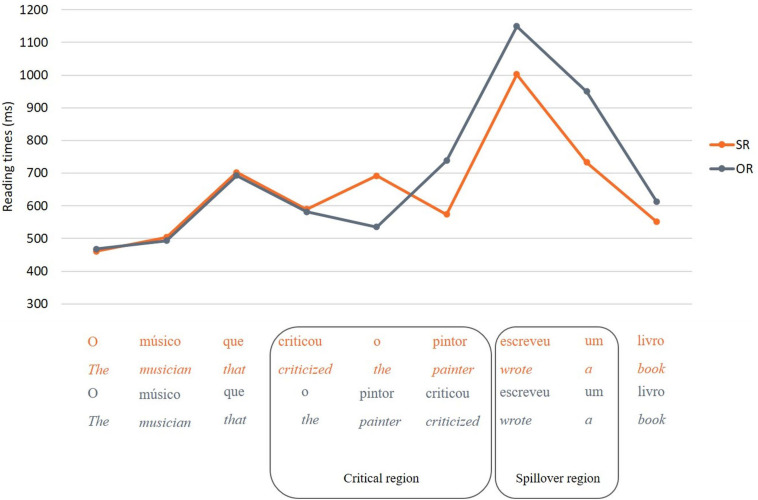
Mean reading times per word for SR and OR conditions.

**FIGURE 7:**
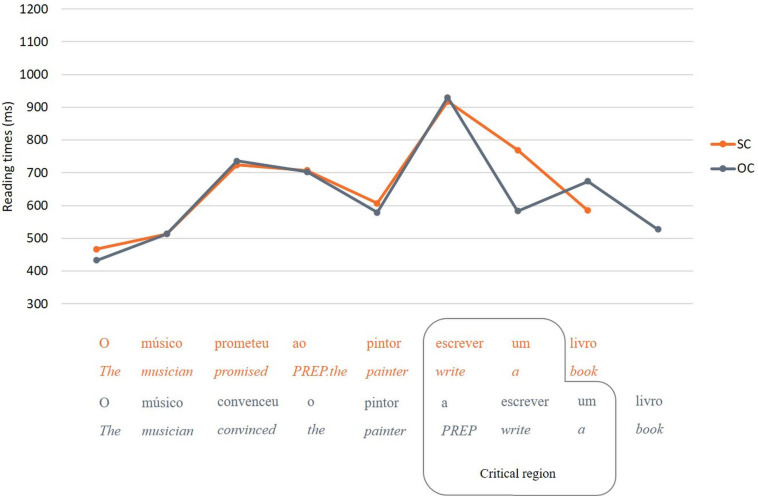
Mean reading times per word for SC and OC conditions.

Figures 6, 7 show the mean reading times per word for relative clause conditions and control conditions, respectively. The dependent variable was the average reading time per word at a given region of interest.

The analysis of relative clause conditions showed that reading times in the critical region were not significantly different between SR and OR sentences, *F*(1, 1689) = 0.51, *p* = 0.475. However, there was a significant difference in the post-critical region, *F*(1, 1706) = 48.98, *p* < 0.001, with longer reading times for OR than for SR (see Figure 6). A follow-up model showed that this difference was not modulated by supplementary tasks^16^.

The model for control conditions revealed significant differences in the critical region, *F*(1, 1639) = 65.31, *p* < 0.001, with longer reading times for SC sentences than for OC sentences^17^ (see Figure 7). A follow-up model showed that this difference was not modulated by supplementary tasks.

## Discussion

We begin by discussing the results from the sentence comprehension task, which bear on the question of whether subject control parallels object relatives in terms of processing complexity and, consequently, on whether processing data warrants a movement analysis of control. Then, we take up the results from the analyses of individual differences in sentence processing and discuss the role of general and syntax-specific processes in intervention.

The results concerning relative clause processing in the sentence comprehension task replicated previous research, revealing complexity effects of OR both offline and online. Offline, we found lower accuracy and higher response times in answering comprehension probes about OR sentences than about SR sentences. Online, we found that OR sentences presented longer reading times than SR sentences in the spillover region. These results indicate that our methodology was sound and provides a valid baseline against which we can compare processing of control.

Whereas the results for relative clauses in the sentence comprehension task were consistent across all measures, the results for control structures were not: (i) there was no significant difference between SC and OC in terms of accuracy in responding to the comprehension probes; (ii) response times were longer for OC than for SC, showing that participants need more time to respond correctly to comprehension questions related to OC sentences; and (iii) there were longer reading times for SC than for OC at the critical region, indicating greater processing demands in reading SC sentences. Taken together, these results show that relative clauses and control structures do not reveal parallel complexity asymmetries. In other words, whereas object relative clauses reveal intervention effects during processing, subject control structures do not. To this extent, these data (processing data from non-brain-damaged adults) do not support a movement approach to control.

Although we did not find evidence for a consistent processing asymmetry between SC and OC, thus warranting the conclusion that the processes underlying comprehension of subject control and object relatives are not of the same nature, we found evidence for greater difficulty with OC in response times and with SC in reading times. We briefly discuss these results in turn.

The longer response times to the probe question observed for OC were unexpected. Here, a suggestion put forward by Boland et al. (1990) may prove relevant. On their view, OC may incur larger processing costs than SC due to a more complex semantic structure: whereas constructing an event structure of a SC verb requires representing a single *doer* involved in two related events, i.e., that denoted by the matrix verb and that denoted by the embedded verb, constructing an event structure of an OC verb requires representing two *doers*, one for each event, and an additional relationship, since the matrix subject is responsible for the event denoted by the embedded verb. Boland et al.’s suggestion is particularly interesting since it places the burden of the complexity effects observed in OC in semantics. Several authors have explored the role of lexical semantics in theories of controller choice, i.e., theories explaining why a verb is an object or a subject control verb (see the summary in Landau, 2013: 125–136), reaching generalizations which a purely syntactic account such as the MTC ignores. Importantly, an MTC account of control cannot offer an explanation for a complexity effect of OC, since OC, which is directly explained by locality principles, is seen as the default control reading in ditransitive structures. If Boland et al. (1990) are on the right track, we must explain why this greater semantic complexity of OC would not affect accuracy and, especially, reading times, but could manifest itself in response times. In what follows, when discussing OR results, we suggest that response times reflect access to a previously encoded semantic representation (which is required to answer the comprehension probe). These issues certainly require further investigation.

The longer reading times in the critical region with SC replicated previous research (Betancort et al., 2006), suggesting that there is an early processing cost in parsing sentences involving SC dependencies. It may be, as Betancort et al. suggest, that two constraints contribute for selection of the controller DP: lexical semantics (i.e., verb meaning, determining correct interpretation) and recency. As in SC these constraints compete, a processing cost might be expected. Nevertheless, the results for reading times should be interpreted with caution, due to the occurrence of an extra preposition in the critical region of OC sentences in European Portuguese. Since the same processing steps (i.e., filling the empty position and integrating it with the verb) are distributed by a longer region, potential slowdowns for OC may be expected to dissipate.

Having established that intervention effects in object relatives are not paralleled in subject control, we now turn to the question of the nature of the processes responsible for intervention. The analysis of individual differences in syntactic effects revealed only null results, except for a significant interaction between Reading Span and relative clause type in response times, indicating that working memory capacity had a stronger effect in the processing of OR than in the processing of SR, with higher working memory associated with faster responses for OR. We believe that these data are consistent with previous claims that syntactic processing is carried out by a functionally isolated cognitive system (Caplan and Waters, 1999; James et al., 2018), which does not draw on general cognitive resources. In what follows, we further discuss the issue of modularity in OR processing, in connection with the significant interaction.

We attach special theoretical significance to the interaction between working memory capacity and OR complexity effects emerging in response times, since, as far as we know, previous research concerning individual differences in relative clause processing has not assessed response times to comprehension probes. Given that, in accordance with much work (Caplan and Waters, 1999, 2005; Caplan et al., 2011; James et al., 2018), we failed to find significant interactions between working memory capacity and OR complexity effects emerging in accuracy and reading times, we speculate that response times may reflect processes different from those indexed by accuracy and reading times. We suggest that reading times and accuracy may directly reflect difficulty in constructing the OR movement dependency, due to intervention: whenever underspecification occurs, the construction of an OR syntactic representation is compromised, inducing a cost in reading times and often causing processing breakdown, lowering accuracy. If we further assume that underspecification is dependent on syntax-specific, functionally isolated processing constraints, the lack of interactions between complexity effects of OR in accuracy and reading times and supplementary tasks is explained. Response times, on the other hand, may be sensibly expected to reflect access to a previously correctly encoded/constructed semantic representation, since they were only analyzed for correct trials. If so, we may assume that longer response times with OR than with SR are due to poorer encoding of OR semantic representations (perhaps as a consequence of intervention), and modulatory effects of working memory capacity on OR complexity effects surfacing in response times may be expected, since the poorer representations of OR should be harder to retrieve, rendering maintenance in working memory especially important.

It is important to note, however, that absence of evidence is not evidence for absence; that is, even though we failed to find significant interactions between supplementary tasks and OR complexity effects in accuracy and response times, future studies could potentially reveal such an effect. As further discussed at the end of the next section, only one supplementary task per construct of interest was used, which leaves open the question of whether the same results would be found if we had used other tasks. Nevertheless, our study is in line with extensive literature wherein interactions between relative clause processing and supplementary tasks have been particularly difficult to find, as noted above. Until such interactions are found, Caplan and Waters (1999) proposal remains plausible.

### Significance and Limitations of the Present Study

The conclusions drawn in the previous section are entirely dependent on the assumption that OR complexity effects consist (at least partly) of intervention effects, as proposed in Grillo (2005; 2008; 2009). However, it could be argued that memory considerations alone suffice to predict a processing asymmetry between OR and SR. In fact, memory constraints are independently motivated. Similarity-based accounts of sentence processing, for instance, are not just consistent with well-established principles in the Memory literature (Nairne, 2002; Oberauer and Kliegl, 2006), but supported by syntactic and semantic similarity effects found to show up in the processing of a wide range of dependencies (for reviews, see Van Dyke and Johns, 2012; Jäger et al., 2017), including filler-gap dependencies (Gordon et al., 2001, 2002; Fedorenko et al., 2006; Van Dyke and McElree, 2006), subject-verb dependencies (Van Dyke, 2007; Tan et al., 2017), agreement dependencies (Wagers et al., 2009; Dillon et al., 2013), negative polarity items (Vasishth et al., 2008) and antecedent-reflexive dependencies (Jäger et al., 2020; but see Dillon et al., 2013 and Dillon, 2014). Although it has been argued that similarity effects in structures involving movement across a hierarchically intervening DP, such as OR, can be reduced to intervention effects (on this point, see Friedmann et al., 2009; Adani et al., 2010; Belletti and Rizzi, 2013; but see also Villata, 2017), dispensing with memory accounts altogether is unwarranted, as similarity effects greatly exceed the boundaries of movement^18^.

Therefore, the assumption that RM contributes to OR complexity over and above memory constraints needs to be justified. We believe that the theoretical cost of this assumption is payed off in empirical coverage. Firstly, attributing complexity effects of OR to hierarchical intervention of the subject DP in the movement dependency explains why a processing advantage for SR is still found in languages in which the word order of SR and OR does not differ, such as German (e.g., Vos et al., 2001; Adelt et al., 2017), and for which memory accounts would predict no asymmetry between SR and OR: even though both subject and object movement cross a linearly intervening DP, only object movement crosses a hierarchically intervening DP. Similarly, and even more tellingly, it may explain why OR complexity effects are found in Mandarin, wherein it is the subject, and not the object, that is more distant from the verb (see Vasishth et al., 2013, for the facts showing a SR advantage in Mandarin; Hu et al., 2016), leading memory accounts to predict an opposite asymmetry (i.e., an object advantage, since in these languages it is subject movement, and not object movement, which crosses a linearly intervening DP). Secondly, it explains why we did not find parallel asymmetries between relative clauses and control structures in this study, contrary to what would be predicted by memory accounts, as the similar surface configurations in OR and SC (i.e., configurations of linear intervention) are expected to be equally taxing to working memory.

If this reasoning is on the right track, then the widespread use of data from processing of relative clauses to inform general models of sentence complexity (e.g., based on general principles of memory) may be inappropriate. The reason for this is that, if the minimality account is right, relative clauses are special structures, in that their complexity results primarily from specific grammatical principles, and not from their surface configuration. More generally, these conclusions speak to the need for a closer dialogue between grammatical and processing models of sentence processing. The interaction should be beneficial in both directions: (i) On the one hand, some processing contrasts of complexity may only be adequately explained by postulating different grammatical representations of the structures contrasted – in our view, this is the case of the contrast between relative clauses and control structures; (ii) On the other hand, competition between equally plausible grammatical analyses for a given structure type may be settled by processing data. We believe that the case of control illustrates this latter point: accepting that OR complexity effects are primarily due to RM, our results lead us to reject a movement-based analysis of control.

Before closing the Discussion, we would like to discuss some limitations of our work concerning the choice of supplementary tasks. On the one hand, cognitive tasks are not process pure, which means that each task measures several processes, only some of which may actually be related to the construct of interest. On the other hand, the same construct of interest may be measured by many different tasks. This suggests that significant interactions with a supplementary task may not be due to the construct of interest that is assumed to be measured, and it also means that alternative tasks, with greater validity, could have been used to measure the constructs of interest. To give a concrete example, working memory capacity, as measured by Reading Span tasks and related tasks (operation span, symmetry span, etc.) has been shown to involve processes of resistance to interference and attention (Kane and Engle, 2000; Engle and Kane, 2004; Bunting, 2006; Unsworth, 2010; Unsworth et al., 2014), leading Engle et al. (e.g., Engle and Kane, 2004) to propose that working memory capacity includes a component of executive attention responsible for maintaining task goals activated under conditions of interference and competition, which has been taken to be responsible for correlations between working memory tasks and other complex tasks (e.g., IQ tasks, Bunting, 2006). If we accept that working memory capacity tasks indeed measure a multifaceted construct, interpreting effects of working memory in terms of capacity may not be entirely accurate. Along the same lines, one may ask if the fluency task is the most appropriate measure of lexical access ability. It is possible that other tasks of lexical access, such as lexical decision tasks, provide a more direct measure of efficiency of access to the lexicon. These are pervasive problems in individual differences research, implying that the conclusions drawn from one single study are limited by the number and nature of the tasks assessed. Future research could test whether the results obtained here can be replicated with other supplementary tasks assumed to measure the same constructs.

## Data Availability Statement

The raw data supporting the conclusions of this article will be made available by the authors, without undue reservation.

## Ethics Statement

The studies involving human participants were reviewed and approved by Ethics Committee of the Faculty of Psychology of the University of Lisbon. Written informed consent from the participants’ legal guardian/next of kin was not required to participate in this study in accordance with the national legislation and the institutional requirements.

## Author Contributions

JD collected the data and performed the statistical analyses, wrote the first draft of the manuscript. All authors contributed to the conception and design of the study. All authors contributed to the revision of the manuscript and approved the submitted version.

## Conflict of Interest

The authors declare that the research was conducted in the absence of any commercial or financial relationships that could be construed as a potential conflict of interest.

## Footnotes

1 *Movement* refers to the syntactic operation that yields structures in which two (or more) positions in the syntactic tree are occupied by copies of the same constituent, only one of which is pronounced.

2 *C-command* refers to a structural configuration: a syntactic node A c-commands a syntactic node B iff: i) either B is A’s sister; ii) or A’s sister contains B.

3 Classes are defined on the basis of distributional and semantic considerations.

4 The moved element in relative clauses may be a DP with a relative morpheme in the D position (see Bianchi, 2002).

5 Obligatory control refers to an obligatory interpretive dependency between the null subject of an infinitival embedded clause (the most common case) and a specific argument of the matrix clause (the controller).

6 Most studies involving processing of control structures in adulthood focused on different questions, such as whether control information is delayed in parsing, e.g., Frazier et al. (1983), Boland et al. (1990). Although potentially informative as to whether subject and object control differ in complexity, these studies yielded conflicting results and, importantly, involved complex interactions between control and wh-dependencies, complicating interpretation of the results.

7 Although agreement match was also manipulated, we will focus only on the grammatical sentences, as data from processing of agreement does not bear on our questions. The effects of agreement match did not interact with the effects of control structure.

8 Processing of a word which follows the word that is being fixated.

9 Defined as “an entity that has a spatiotemporal location so that it can later be referred to with an anaphoric expression, such as a pronoun for NPs, or tense on a verb for events” (Gibson, 2000, p. 111).

10 It should be noted that memory models predict parallel processing difficulties in OR and SC independently of Generalized Minimality, and therefore independently of the analysis adopted for Control (i.e., movement vs non-movement). The reason for this is that memory models focus on surface structure, and therefore on linear, and not hierarchical, intervention. We return to this point in the Discussion section, where we elaborate on the relationship between memory-based and syntactically based models of complexity effects.

11 Note, though, that this question is orthogonal to the question of what type of memory constraints influence syntactic processing. It may be, for instance, that syntactic processing is susceptible to capacity overload or similarity-interference, just like general memory systems are thought to be, without interacting with general measures of memory capacity or resistance to interference. In other words, the same type of mechanism may be implemented in both general and particular memory systems (i.e., different memory systems might be viewed as tokens of the same type).

12 One of the reviewers questioned the interpretation of SR sentences, namely whether they are ambiguous between a SR and an OR reading. It is a fact that European Portuguese allows sentences in which the subject follows the verb (VS sentences), as shown by Ambar (1992), and Costa (1998). To this extent, one may ask whether the sentence in (8a) may be interpreted as an OR if the DP [o pintor] is interpreted as a post-verbal subject in the relative clause. According to our intuitions, this is an interpretation which is very difficult to obtain, not unexpectedly if, as Ambar (1992) and Costa (1998) have shown, the VS word order is possible in particular discourse conditions which allow the subject to be focused: it would be difficult to interpret the overt DP within the relative clause in (8a) as focused. Moreover, this type of interpretation should be supported by a rich discourse context and our sentences were tested in out-of-the-blue contexts. Importantly, since this problem would affect SR (and not OR), if there is any ambiguity in the interpretation of this type of item this should negatively affect our results in this condition, in terms of accuracy, reading times and response times. As it will be shown, our results do not suggest that SR items were interpreted as ambiguous.

13 www.psnet.com

14 The minimum value in the Brown-Peterson task deserves a note. Theoretically, participants should not have negative scores, since our dependent measure [(List1-List3)/List1] is presumed to index susceptibility to interference (one cannot be negatively susceptible to interference). Negative scores reflect facilitation across the task, which runs contrary to our expectations, and are thus potentially problematic. However, we note that only one participant had a negative score. Since there was only one negative score, and the group performance was expected, we see no reason to question the validity of our task.

15 The model failed to converge with random effects of subjects and items. Therefore, we included only random effects of subjects.

16 The model failed to converge with random effects of subjects and items. Therefore, we included only random effects of subjects.

17 A *post hoc* analysis revealed no difference between OC structures and SC structures with *ameaçar* “threaten” in reading times at the preposition introducing the subordinated clause, *F*(1, 1070) = 0.021, *p* = 0.886, nor at the embedded verb, *F*(1, 1071) = 0.292, *p* = 0.589. Therefore, it is possible that the occurrence of the extra preposition in SC structures with *ameaçar* led participants to interpret them as OC structures, justifying the atypically low accuracy.

18 On the other hand, it has been proposed that (at least some) island effects should be totally reduced to memory constraints, dispensing with grammatical principles concerned with locality (see Hofmeister and Sag, 2010). We believe that this move is equally unjustified. We point the reader to the discussion in Sprouse and Villata (unpublished) for arguments against a reductionist approach to island phenomena. On the relation between similarity-effects and RM, see also Ortega-Santos (2011), who proposes that RM should be seen as a grammaticized response to memory, thus attempting to ground Minimalist abstract notions such as “computational efficiency” (Chomsky, 2005) in well-established principles of memory functioning.

## References

[B1] Adams A. M. Gathercole S. E. (2000). Limitations in working memory: implications for language development. *Int. J. Lang. Commun. Disord.* 35 95–116. 10.1080/136828200247278 10824227

[B2] Adani F. van der Lely H. K. J. Forgiarini M. Guasti M. T. (2010). Grammatical feature dissimilarities make relative clauses easier: a comprehension study with Italian children. *Lingua* 120 2148–2166. 10.1016/j.lingua.2010.03.018 21151323 PMC2956846

[B3] Adelt A. Stadie N. Lassotta R. Adani F. Burchert F. (2017). Feature dissimilarities in the processing of German relative clauses in aphasia. *J. Neurolinguistics* 44 17–37. 10.1016/j.jneuroling.2017.01.002

[B4] Agostinho C. Santos A. L. Duarte I. (2018). “The acquisition of control in European Portuguese,” in *Complement Clauses in Portuguese: Syntax and Acquisition*, eds Santos A. L. Gonçalves A. (Amsterdam: John Benjamins), 261–294.

[B5] Ambar M. (1992). *Para Uma Sintaxe da Inversão Sujeito-Verbo Em Português.* Lisboa: Edições Colibri.

[B6] Archibald L. M. (2017). Working memory and language learning: a review. *Child Lang. Teach. Ther.* 33 5–17. 10.1177/0265659016654206

[B7] Baayen R. H. Davidson D. J. Bates D. M. (2008). Mixed-effects modeling with crossed random effects for subjects and items. *J. Mem. Lang.* 59 390–412. 10.1016/j.jml.2007.12.005

[B8] Baayen R. H. Milin P. (2010). Analyzing reaction times. *Int. J. Psychol. Res.* 3 12–28. 10.21500/20112084.807

[B9] Baddeley A. (2012). Working memory: theories, models, and controversies. *Annu. Rev. Psychol.* 63 1–29. 10.1146/annurev-psych-120710-100422 21961947

[B10] Belletti A. Rizzi L. (2013). “Intervention in grammar and processing,” in *From Grammar to Meaning*, eds Caponigro I. Cecchetto C. (Cambridge: Cambridge University Press), 294–311.

[B11] Betancort M. Carreiras M. Acuña-Fariña C. (2006). Processing controlled PROs in Spanish. *Cognition* 100 217–282. 10.1016/j.cognition.2005.04.001 16310761

[B12] Bianchi V. (2002). Headed relative clauses in generative syntax – Part I. *Glot Int.* 6 197–204.

[B13] Boeckx C. Hornstein N. Nunes J. (2010). *Control as Movement.* Cambridge: Cambridge University Press.

[B14] Boland J. E. Tanenhaus M. K. Garnsey S. M. (1990). Evidence for the immediate use of verb control information in sentence processing. *J. Mem. Lang.* 29 413–432. 10.1016/0749-596X(90)90064-7

[B15] Borella E. Carretti B. Mammarella I. (2006). Do working memory and susceptibility to interference predict individual differences in fluid intelligence? *Eur. J. Cogn. Psychol.* 18 51–69. 10.1080/09541440500215962

[B16] Bunting M. (2006). Proactive interference and item similarity in working memory. *J. Exp. Psychol. Learn. Mem. Cogn.* 32 183–196. 10.1037/0278-7393.32.2.183 16569140

[B17] Caplan D. DeDe G. Waters G. Michaud J. Tripodis Y. (2011). Effects of age, speed of processing, and working memory on comprehension of sentences with relative clauses. *Psychol. Aging* 26 439–450. 10.1037/a0021837 21480714

[B18] Caplan D. Waters G. (2005). The relationship between age, processing speed, working memory capacity, and language comprehension. *Memory* 13 403–413. 10.1080/09658210344000459 15952262

[B19] Caplan D. Waters G. S. (1999). Verbal working memory and sentence comprehension. *Behav. Brain Sci.* 22 77–94. 10.1017/S0140525X99001788 11301522

[B20] Caramazza A. Zurif E. B. (1976). Dissociation of algorithmic and heuristic processes in language comprehension: evidence from aphasia. *Brain Lang.* 3 572–582. 10.1016/0093-934X(76)90048-1 974731

[B21] Chomsky C. (1969). *The Acquisition of Syntax in Children from 5 to 10*, 1972 Edn Cambridge, MA: The MIT Press.

[B22] Chomsky N. (1981). *Lectures on Government and Binding: The Pisa Lectures*, (3rd revised edition) Edn Dordrecht: Foris Publications.

[B23] Chomsky N. (2005). Three factors in language design. *Linguistic Inquiry* 36 1–22. 10.1162/0024389052993655

[B24] Cilibrasi L. Adani F. Tsimpli I. (2019). Reading as a predictor of complex syntax. The case of relative clauses. *Front. Psychol.* 10:1480. 10.3389/fpsyg.2019.01450 31354557 PMC6635578

[B25] Clark D. G. Kapur P. Geldmacher D. S. Brockington J. C. Harrell L. DeRamus T. P. (2014). Latent information in fluency lists predicts functional decline in persons at risk for Alzheimer disease. *Cortex* 55 202–218. 10.1016/j.cortex.2013.12.013 24556551 PMC4039569

[B26] Conway A. R. A. Kane M. J. Bunting M. F. Hambrick D. Z. Wilhelm O. Engle R. W. (2005). Working memory span tasks: a methodological review and user’s guide. *Psychon. Bull. Rev.* 12 769–786. 10.3758/BF03196772 16523997

[B27] Costa J. (1998). *Word Order Variation. A Constraint-based Approach.* Doctoral dissertation, HIL / Leiden University, Leiden.

[B28] Costa J. Grillo N. Lobo M. (2012). Minimality beyond lexical restrictions: processing and acquisition of free wh-dependencies in European Portuguese. *Rev. Roumaine de Linguistique* 57 143–160.

[B29] Costa J. Lobo M. Silva C. (2011). Subject–object asymmetries in the acquisition of Portuguese relative clauses: adults vs. children. *Lingua* 121 1083–1100. 10.1016/j.lingua.2011.02.001

[B30] Culicover P. W. Jackendoff R. (2001). Control is not movement. *Linguistic Inquiry* 32 493–512. 10.1162/002438901750372531

[B31] Culicover P. W. Jackendoff R. (2006). Turn over control to the semantics! *Syntax* 9 131–152. 10.1111/j.1467-9612.2006.00085.x

[B32] Daneman M. Carpenter P. A. (1980). Individual differences in working memory and reading. *J. Verbal Learn. Verbal Behav.* 19 450–466. 10.1016/S0022-5371(80)90312-6

[B33] Dillon B. (2014). Syntactic memory in the comprehension of reflexive dependencies: an overview: syntactic memory and reflexives. *Lang. Linguistics Compass* 8 171–187. 10.1111/lnc3.12075

[B34] Dillon B. Mishler A. Sloggett S. Phillips C. (2013). Contrasting intrusion profiles for agreement and anaphora: experimental and modeling evidence. *J. Mem. Lang.* 69 85–103. 10.1016/j.jml.2013.04.003

[B35] Engle R. W. Kane M. J. (2004). Executive attention, working memory capacity, and a two-factor theory of cognitive control. *Psychol. Learn. Motiv.* 44 145–200. 10.1016/S0079-7421(03)44005-X

[B36] Fedorenko E. Gibson E. Rohde D. (2006). The nature of working memory capacity in sentence comprehension: evidence against domain-specific working memory resources. *J. Mem. Lang.* 54 541–553. 10.1016/j.jml.2005.12.006

[B37] Foraker S. McElree B. (2011). Comprehension of linguistic dependencies: speed-accuracy tradeoff evidence for direct-access retrieval from memory: comprehending dependencies. *Lang. Linguistics Compass* 5 764–783. 10.1111/j.1749-818X.2011.00313.x 22448181 PMC3310376

[B38] Frazier L. Clifton C. Randall J. (1983). Filling gaps: decision principles and structure in sentence comprehension. *Cognition* 13 187–222. 10.1016/0010-0277(83)90022-7

[B39] Friedman N. P. Miyake A. (2004). The relations among inhibition and interference control functions: a latent-variable analysis. *J. Exp. Psychol. Gen.* 133 101–135. 10.1037/0096-3445.133.1.101 14979754

[B40] Friedmann N. Belletti A. Rizzi L. (2009). Relativized relatives: types of intervention in the acquisition of A-bar dependencies. *Lingua* 119 67–88. 10.1016/j.lingua.2008.09.002

[B41] Gibson E. (1998). Linguistic complexity: locality of syntactic dependencies. *Cognition* 68 1–76. 10.1016/S0010-0277(98)00034-1 9775516

[B42] Gibson E. (2000). “The dependency locality theory: a distance-based theory of linguistic complexity,” in *Image, Language, Brain: Papers From the First Mind Articulation Project Symposium*, eds Marantz A. Miyashita Y. O’Neil W. (Cambridge, MA: The MIT Press), 94–126.

[B43] Gonçalves R. (2015). Romance languages do not have double objects: evidence from European Portuguese and Spanish. *Estudos de Lingüistica Galega* 7 53–67. 10.15304/elg.7.2337

[B44] Gordon P. C. Hendrick R. Johnson M. (2001). Memory interference during language processing. *J. Exp. Psychol. Learn. Mem. Cogn.* 27 1411–1423. 10.1037//0278-7393.27.6.1411 11713876

[B45] Gordon P. C. Hendrick R. Johnson M. (2004). Effects of noun phrase type on sentence complexity. *J. Mem. Lang.* 51 97–114. 10.1016/j.jml.2004.02.003

[B46] Gordon P. C. Hendrick R. Levine W. H. (2002). Memory-load interference in syntactic processing. *Psychol. Sci.* 13 425–430. 10.1111/1467-9280.00475 12219808

[B47] Grillo N. (2005). “Minimality effects in agrammatic comprehension,” in *Proceedings of ConSOLE XIII*, eds Blaho S. Vicente L. Schoorlemmer E. (Leiden: Leiden University), 107–120.

[B48] Grillo N. (2008). *Generalized Minimality: Syntactic Underspecification in Broca’s Aphasia.* Doctoral dissertation University of Utrecht, Utrecht.

[B49] Grillo N. (2009). Generalized minimality: feature impoverishment and comprehension deficits in agrammatism. *Lingua* 119 1426–1443. 10.1016/j.lingua.2008.04.003

[B50] Grodzinsky Y. (1984). The syntactic characterization of agrammatism. *Cognition* 16 99–120. 10.1016/0010-0277(84)90001-5 6205816

[B51] Grodzinsky Y. (2000). The neurology of syntax: language use without Broca’s area. *Behav. Brain Sci.* 23 1–21. 10.1017/S0140525X00002399 11303337

[B52] Hofmeister P. Sag I. A. (2010). Cognitive constraints and island effects. *Language* 86 366–415. 10.1353/lan.0.0223 22661792 PMC3364522

[B53] Hofmeister P. Vasishth S. (2014). Distinctiveness and encoding effects in online sentence comprehension. *Front. Psychol.* 5:1237. 10.3389/fpsyg.2014.01237 25566105 PMC4264409

[B54] Hornstein N. (1999). Movement and control. *Linguistic Inquiry* 30 69–96. 10.1162/0024389041402625

[B55] Hornstein N. Polinsky M. (2010). “Control as movement: across languages and constructions,” in *Movement Theory of Control*, eds Hornstein N. Polinsky M. (Philadelphia, PA: John Benjamins), 1–41.

[B56] Hu S. Gavarró A. Guasti M. T. (2016). Children’s production of head-final relative clauses: the case of Mandarin. *Appl. Psycholinguistics* 37 323–346. 10.1017/S0142716414000587

[B57] Jaeger T. F. (2008). Categorical data analysis: away from ANOVAs (transformation or not) and towards logit mixed models. *J. Mem. Lang.* 59 434–446. 10.1016/j.jml.2007.11.007 19884961 PMC2613284

[B58] Jäger L. A. Engelmann F. Vasishth S. (2017). Similarity-based interference in sentence comprehension: literature review and Bayesian meta-analysis. *J. Mem. Lang.* 94 316–339. 10.1016/j.jml.2017.01.004

[B59] Jäger L. A. Mertzen D. Van Dyke J. A. Vasishth S. (2020). Interference patterns in subject-verb agreement and reflexives revisited: a large-sample study. *J. Mem. Lang.* 111:104063. 10.1016/j.jml.2019.104063 33100507 PMC7583648

[B60] James A. N. Fraundorf S. H. Lee E.-K. Watson D. G. (2018). Individual differences in syntactic processing: is there evidence for reader-text interactions? *J. Mem. Lang.* 102 155–181. 10.1016/j.jml.2018.05.006 30713367 PMC6350810

[B61] Just M. A. Carpenter P. A. (1980). A theory of reading: from eye fixations to comprehension. *Psychol. Rev.* 87 329–354. 10.1037/0033-295X.87.4.329 7413885

[B62] Just M. A. Carpenter P. A. (1992). A capacity theory of comprehension: individual differences in working memory. *Psychol. Rev.* 99 122–148. 10.1037/0033-295X.99.1.122 1546114

[B63] Just M. A. Carpenter P. A. Woolley J. D. (1982). Paradigms and processes in reading comprehension. *J. Exp. Psychol. Gen.* 111 228–238. 10.1037/0096-3445.111.2.228 6213735

[B64] Kane M. J. Engle R. W. (2000). Working-memory capacity, proactive interference, and divided attention: limits on long-term memory retrieval. *J. Exp. Psychol. Learn. Mem. Cogn.* 26 336–358. 10.1037/0278-7393.26.2.336 10764100

[B65] Kane M. J. Hambrick D. Z. Tuholski S. W. Wilhelm O. Payne T. W. Engle R. W. (2004). The generality of working memory capacity: a latent-variable approach to verbal and visuospatial memory span and reasoning. *J. Exp. Psychol. Gen.* 133 189–217. 10.1037/0096-3445.133.2.189 15149250

[B66] Kayne R. S. (1994). *The Antisymmetry of Syntax.* Cambridge, MA: The MIT Press.

[B67] King J. Just M. A. (1991). Individual differences in syntactic processing: the role of working memory. *J. Mem. Lang.* 30 580–602. 10.1016/0749-596X(91)90027-H

[B68] Kirby S. Davies W. D. Dubinsky S. (2010). Up to D[eb]ate on raising and control Part 1: properties and analyses of the constructions. *Lang. Linguistics Compass* 4 390–400. 10.1111/j.1749-818X.2010.00198.x

[B69] Kwon N. Kluender R. Kutas M. Polinsky M. (2013). Subject/object processing asymmetries in Korean relative clauses: evidence from ERP data. *Language* 89 537–585. 10.1353/lan.2013.0044 25400303 PMC4231604

[B70] Kwon N. Sturt P. (2016). Processing control information in a nominal control construction: an eye-tracking study. *J. Psycholinguistic Res.* 45 779–793. 10.1007/s10936-015-9374-2 25980968

[B71] Landau I. (2003). Movement out of control. *Linguistic Inquiry* 34 471–498. 10.1162/002438903322247560

[B72] Landau I. (2013). *Control in Generative Grammar. A Research Companion.* Cambridge: Cambridge University Press.

[B73] Larson R. (1988). On the double object construction. *Linguistic Inquiry* 19 335–391.

[B74] Lewis R. L. Vasishth S. Van Dyke J. A. (2006). Computational principles of working memory in sentence comprehension. *Trends Cogn. Sci.* 10 447–454. 10.1016/j.tics.2006.08.007 16949330 PMC2239011

[B75] Lobo M. Santos A. L. Soares-Jesel C. (2016). Syntactic structure and information structure: the acquisition of Portuguese clefts and be-fragments. *Lang. Acquisition* 23 142–174. 10.1080/10489223.2015.1067317

[B76] Locker L. Hoffman L. Bovaird J. A. (2007). On the use of multilevel modeling as an alternative to items analysis in psycholinguistic research. *Behav. Res. Methods* 39 723–730. 10.3758/BF03192962 18183884

[B77] Martins A. Santos A. L. Duarte I. (2018). “Comprehension of relative clauses vs. control structures in SLI and ASD children,” in *BUCLD 42: Proceedings of the 42nd annual Boston University Conference on Language Development*, eds Bertolini A. B. Kaplan M. J. (Somerville, MA: Cascadilla Press), 493–506.

[B78] Mateu Martin V. E. (2016). *Intervention Effects in the Acquisition of Raising and Control: Evidence from English and Spanish.* Doctoral dissertation, UCLA, Los Angeles, CA.

[B79] McElree B. Foraker S. Dyer L. (2003). Memory structures that subserve sentence comprehension. *J. Mem. Lang.* 48 67–91. 10.1016/S0749-596X(02)00515-6

[B80] Nairne J. S. (2002). Remembering over the short-term: the case against the standard model. *Annu. Rev. Psychol.* 53 53–81. 10.1146/annurev.psych.53.100901.135131 11752479

[B81] Ness T. Meltzer-Asscher A. (2017). Working memory in the processing of long-distance dependencies: interference and filler maintenance. *J. Psycholinguistic Res.* 46 1353–1365. 10.1007/s10936-017-9499-6 28528512

[B82] Nicenboim B. Vasishth S. Gattei C. Sigman M. Kliegl R. (2015). Working memory differences in long-distance dependency resolution. *Front. Psychol.* 6:312. 10.3389/fpsyg.2015.00312 25852623 PMC4369666

[B83] Nicol J. Swinney D. (1989). The role of structure in coreference assignment during sentence comprehension. *J. Psycholinguistic Res.* 18 5–19. 10.1007/BF01069043 2647962

[B84] Oberauer K. Kliegl R. (2006). A formal model of capacity limits in working memory. *J. Mem. Lang.* 55 601–626. 10.1016/j.jml.2006.08.009

[B85] Ortega-Santos I. (2011). On Relativized Minimality, memory and cue-based parsing. *IBERIA Int. J. Theor. Linguistics* 3 35–64.

[B86] Peirce J. W. Gray J. R. Simpson S. MacAskill M. R. Höchenberger R. Sogo H. (2019). PsychoPy2: experiments in behavior made easy. *Behav. Res. Methods* 51 195–203. 10.3758/s13428-018-01193-y 30734206 PMC6420413

[B87] Pettigrew C. Martin R. C. (2014). Cognitive declines in healthy aging: evidence from multiple aspects of interference resolution. *Psychol. Aging* 29 187–204. 10.1037/a0036085 24955989

[B88] Pinto A. C. (1992). *Categorizacão de Itens Verbais: Medidas de Frequência de Producão e de Tipicidade [Categorization of Verbal Items: Production Frequency and Typicality Measures] (Tech. Rep.).* Porto: Faculdade de Psicologia e de Ciências da Educacão da Universidade do Porto.

[B89] Rizzi L. (1990). *Relativized Minimality.* Cambridge, MA: The MIT Press.

[B90] Rizzi L. (2004). “Locality and left periphery,” in *Structures and Beyond: The Cartography of Syntactic Structures*, ed. Belletti A. (New York, NY: Oxford University Press), 223–251.

[B91] Rosen V. M. Engle R. W. (1997). The role of working memory capacity in retrieval. *J. Exp. Psychol. Gen.* 126 211–227. 10.1037/0096-3445.126.3.211 9281831

[B92] Tan Y. Martin R. C. Van Dyke J. A. (2017). Semantic and syntactic interference in sentence comprehension: a comparison of working memory models. *Front. Psychol.* 8:198. 10.3389/fpsyg.2017.00198 28261133 PMC5309252

[B93] Troyer A. K. Moscovitch M. (2006). “Cognitive processes of verbal fluency tasks,” in *Studies on Neuropsychology, Neurology and Cognition. The Quantified Process Approach to Neuropsychological Assessment*, ed. Poreh A. M. (Philadelphia, PA: Taylor and Francis), 143–160.

[B94] Troyer A. K. Moscovitch M. Winocur G. (1997). Clustering and switching as two components of verbal fluency: evidence from younger and older healthy adults. *Neuropsychology* 11:138. 10.1037/0894-4105.11.1.138 9055277

[B95] Unsworth N. (2010). Interference control, working memory capacity, and cognitive abilities: a latent variable analysis. *Intelligence* 38 255–267. 10.1016/j.intell.2009.12.003

[B96] Unsworth N. Fukuda K. Awh E. Vogel E. K. (2014). Working memory and fluid intelligence: capacity, attention control, and secondary memory retrieval. *Cogn. Psychol.* 71 1–26. 10.1016/j.cogpsych.2014.01.003 24531497 PMC4484859

[B97] Van Dyke J. A. (2007). Interference effects from grammatically unavailable constituents during sentence processing. *J. Exp. Psychol. Learn. Mem. Cogn.* 33 407–430. 10.1037/0278-7393.33.2.407 17352621 PMC2077343

[B98] Van Dyke J. A. Johns C. L. (2012). Memory interference as a determinant of language comprehension. *Lang. Linguistics Compass* 6 193–211. 10.1002/lnc3.330 22773927 PMC3389825

[B99] Van Dyke J. A. Johns C. L. Kukona A. (2014). Low working memory capacity is only spuriously related to poor reading comprehension. *Cognition* 131 373–403. 10.1016/j.cognition.2014.01.007 24657820 PMC3988267

[B100] Van Dyke J. A. McElree B. (2006). Retrieval interference in sentence comprehension. *J. Mem. Lang.* 55 157–166. 10.1016/j.jml.2006.03.007 18209744 PMC2206541

[B101] Vasishth S. Brussow S. Lewis R. Drenhaus H. (2008). Processing polarity: how the ungrammatical intrudes on the grammatical. *Cogn. Sci. Multidiscip. J.* 32 685–712. 10.1080/03640210802066865 21635350

[B102] Vasishth S. Chen Z. Li Q. Guo G. (2013). Processing Chinese relative clauses: evidence for the subject-relative advantage. *PLoS One* 8:e77006. 10.1371/journal.pone.0077006 24098575 PMC3788747

[B103] Vergnaud J. R. (1974). *French Relative Clauses.* Doctoral dissertation, MIT, Cambridge, MA.

[B104] Villata S. (2017). *Intervention Effects in Sentence Processing.* Doctoral dissertation, University of Geneva, Geneva.

[B105] Vos S. H. Gunter T. C. Schriefers H. Friederici A. D. (2001). Syntactic parsing and working memory: the effects of syntactic complexity, reading span, and concurrent load. *Lang. Cogn. Process.* 16 65–103. 10.1080/01690960042000085

[B106] Wagers M. W. Lau E. F. Phillips C. (2009). Agreement attraction in comprehension: representations and processes. *J. Mem. Lang.* 61 206–237. 10.1016/j.jml.2009.04.002

[B107] Wagers M. W. Phillips C. (2014). Going the distance: memory and control processes in active dependency construction. *Quarterly J. Exp. Psychol.* 67 1274–1304. 10.1080/17470218.2013.858363 24320939

[B108] Wechsler D. (2008). *Escala de Inteligência de Wechsler para Adultos – 3° Edição (WAIS-III).* Lisboa: Cegoc.

[B109] Zurif E. Swinney D. Prather P. Solomon J. Bushell C. (1993). An on-line analysis of syntactic processing in Broca’s and Wernicke’s aphasia. *Brain Lang.* 45 448–464. 10.1006/brln.1993.1054 8269334

