# Supplementary material for: Assessing Intervention Effects in Sentence Processing: Object Relatives vs. Subject Control
Source: Front Psychol. 2021 Feb 2;12:610909. doi: 10.3389/fpsyg.2021.610909 (PMC7884622; doi:10.3389/fpsyg.2021.610909)
Supplement: Supplementary file 1 [file Table_1.DOCX]

Supplementary Material

## Appendix I. Experimental sets used in the sentence comprehension task. Translations are intended to conserve meaning. Sentence structure is not always preserved in the translation (for a thorough description of the structure of the sentences used in the present study, see *Sentence comprehension task* section in *Materials and Procedure*). a = subject relative, b = object relative, c = subject control, d = object control.

1. a. O político que ofendeu o barbeiro escalou a montanha

‘The politician that offended the barber climbed the mountain’

b. O político que o barbeiro ofendeu escalou a montanha

‘The politician that the barber offended climbed the mountain’

c. O político jurou ao barbeiro escalar a montanha

‘The politician swore to the barber to climb the mountain’

d. O político encorajou o barbeiro a escalar a montanha

‘The politician encouraged the barber to climb the mountain’

2. a. O linguista que salvou o palhaço vendeu os quadros

‘The linguist that saved the clown sold the paintings’

b. O linguista que o palhaço salvou vendeu os quadros

‘The linguist that the clown saved sold the paintings’

c. O linguista jurou ao palhaço vender os quadros

‘The linguist swore to the clown to sell the paintings’

d. O linguista encorajou o palhaço a vender os quadros

‘The linguist encouraged the clown to sell the paintings’

3. a. A historiadora que confrontou a artista rejeitou a oferta

‘The historian that confronted the artist rejected the offer’

b. A historiadora que a artista confrontou rejeitou a oferta

‘The historian that the artist confronted rejected the offer’

c. A historiadora jurou à artista rejeitar a oferta

‘The historian swore to the artist to reject the offer’

d. A historiadora encorajou a artista a rejeitar a oferta

‘The historian encouraged the artist to reject the offer’

4. a. O canalizador que agrediu o motorista visitou o palácio

‘The plumber that assaulted the driver visited the palace’

b. O canalizador que o motorista agrediu visitou o palácio

‘The plumber that the driver assaulted visited the palace’

c. O canalizador jurou ao motorista visitar o palácio

‘The plumber swore to the driver to visit the palace’

d. O canalizador encorajou o motorista a visitar o palácio

‘The plumber encouraged the driver to visit the palace’

5. a. A tradutora que reconheceu a analista desenhou o logotipo

‘The translator that recognized the analyst drew the logotype’

b. A tradutora que a analista reconheceu desenhou o logotipo

‘The translator that the analyst recognized drew the logotype’

c. A tradutora jurou à analista desenhar o logotipo

‘The translator swore to the analyst to draw the logotype’

d. A tradutora encorajou a analista a desenhar o logotipo

‘The translator encouraged the analyst to draw the logotype’

6. a. O informático que assustou o físico apresentou o espetáculo

‘The computer technician that scared the physicist presented the show’

b. O informático que o físico assustou apresentou o espetáculo

‘The computer technician that the physicist scared presented the show’

c. O informático jurou ao físico apresentar o espetáculo

‘The computer technician swore to the physicist to present the show’

d. O informático encorajou o físico a apresentar o espetáculo

‘The computer technician encouraged the physicist to present the show’

7. a. O bailarino que divertiu o repórter cozinhou o bife

‘The dancer that amused the reporter cooked the steak’

b. O bailarino que o repórter divertiu cozinhou o bife

‘The dancer that the reporter amused cooked the steak’

c. O bailarino assegurou ao repórter cozinhar o bife

‘The dancer assured the reporter that he would cook the steak’

d. O bailarino obrigou o repórter a cozinhar o bife

‘The dancer forced the reporter to cook the steak’

8. a. O guitarrista que inspirou o jogador procurou o artigo

‘The guitarist that inspired the player searched for the article’

b. O guitarrista que o jogador inspirou procurou o artigo

‘The guitarist that the player inspired searched for the article’

c. O guitarrista assegurou ao jogador procurar o artigo

‘The guitarist assured the player that he would search for the article’

d. O guitarrista obrigou o jogador a procurar o artigo

‘The guitarist forced the player to search for the article’

9. a. A psicóloga que chamou a estilista imprimiu os documentos

‘The psychologist that called the stylist printed the documents’

b. A psicóloga que a estilista chamou imprimiu os documentos

‘The psychologist that the stylist called printed the documents’

c. A psicóloga assegurou à estilista imprimir os documentos

‘The psychologist assured the stylist that she would print the documents’

d. A psicóloga obrigou a estilista a imprimir os documentos

‘The psychologist forced the stylist to print the documents’

10. a. O eletricista que abraçou o carteiro recolheu as assinaturas

‘The electrician that hugged the mailman collected the signatures’

b. O eletricista que o carteiro abraçou recolheu as assinaturas

‘The electrician that the mailman hugged collected the signatures’

c. O eletricista assegurou ao carteiro recolher as assinaturas

‘The electrician assured the mailman that he would collect the signatures’

d. O eletricista obrigou o carteiro a recolher as assinaturas

‘The electrician forced the mailman to collect the signatures’

11. a. A cientista que protegeu a escritora planeou a viagem

‘The scientist that protected the writer planned the trip’

b. A cientista que a escritora protegeu planeou a viagem

‘The scientist that the writer protected planned the trip’

c. A cientista assegurou à escritora planear a viagem

‘The scientist assured the writer that she would plan the trip’

d. A cientista obrigou a escritora a planear a viagem

‘The scientist forced the writer to plan the trip’

12. a. O engenheiro que irritou o treinador enviou a mensagem

‘The engineer that annoyed the trainer sent the message’

b. O engenheiro que o treinador irritou enviou a mensagem

‘The engineer that the trainer annoyed sent the message’

c. O engenheiro assegurou ao treinador enviar a mensagem

‘The engineer assured the trainer that he would send the message’

d. O engenheiro obrigou o treinador a enviar a mensagem

‘The engineer forced the trainer to send the message’

13. a. O matemático que chateou o bombeiro escondeu o presente

‘The mathematician that upset the fire-fighter hid the gift’

b. O matemático que o bombeiro chateou escondeu o presente

‘The mathematician that the fire-fighter upset hid the gift’

c. O matemático ameaçou o bombeiro de esconder o presente

‘The mathematician threatened the fire-fighter with hiding the gift’

d. O matemático autorizou o bombeiro a esconder o presente

‘The mathematician authorized the fire-fighter to hide the gift’

14. a. A violinista que confortou a médica abandonou o grupo

‘The violinist that comforted the doctor abandoned the group’

b. A violinista que a médica confortou abandonou o grupo

‘The violinist that the doctor comforted abandoned the group’

c. A violinista ameaçou a médica de abandonar o grupo

‘The violinist threatened the doctor with abandoning the group’

d. A violinista autorizou a médica a abandonar o grupo

‘The violist authorized the doctor to abandon the group’

15. a. A atriz que corrigiu a ciclista trancou a porta

‘The actress that corrected the cyclist locked the door’

b. A atriz que a ciclista corrigiu trancou a porta

‘The actress that the cyclist corrected locked the door’

c. A atriz ameaçou a ciclista de trancar a porta

‘The actress threatened the cyclist with locking the door’

d. A atriz autorizou a ciclista a trancar a porta

‘The actress authorized the cyclist to lock the door’

16. a. A veterinária que atacou a educadora contactou as finanças

‘The veterinarian that attacked the preschool teacher contacted the financial authority’

b. A veterinária que a educadora atacou contactou as finanças

‘The veterinarian that the preschool teacher attacked contacted the financial authority’

c. A veterinária ameaçou a educadora de contactar as finanças

‘The veterinarian threatened the preschool teacher with contacting the financial authority’

d. A veterinária autorizou a educadora a contactar as finanças

‘The veterinarian authorized the preschool teacher to contact the financial authority’

17. a. A cabeleireira que motivou a dentista apagou os ficheiros

‘The hairdresser that motivated the dentist deleted the files’

b. A cabeleireira que a dentista motivou apagou os ficheiros

‘The hairdresser that the dentist motivated deleted the files’

c. A cabeleireira ameaçou a dentista de apagar os ficheiros

‘The hairdresser threatened the dentist with deleting the files’

d. A cabeleireira autorizou a dentista a apagar os ficheiros

‘The hairdresser authorized the dentist to delete the files’

18. a. A jornalista que perdoou a terapeuta partiu o copo

‘The journalist that forgave the therapist broke the glass’

b. A jornalista que a terapeuta perdoou partiu o copo

‘The journalist that the therapist forgave broke the glass’

c. A jornalista ameaçou a terapeuta de partir o copo

‘The journalist threatened the therapist with breaking the glass’

d. A jornalista autorizou a terapeuta a partir o copo

‘The journalist authorized the therapist to break the glass’

19. a. A agricultora que consolou a bióloga vestiu o casaco

‘The farmer that comforted the biologist put on the coat’

b. A agricultora que a bióloga consolou vestiu o casaco

‘The farmer that the biologist comforted put on the coat’

c. A agricultora garantiu à bióloga vestir o casaco

‘The farmer assured the biologist that she would put on the coat’

d. A agricultora forçou a bióloga a vestir o casaco

‘The farmer forced the biologist to put on the coat’

20. a. O poeta que distraiu o geólogo atendeu o telefone

‘The poet that distracted the geologist answered the telephone’

b. O poeta que o geólogo distraiu atendeu o telefone

‘The poet that the geologist distracted answered the telephone’

c. O poeta garantiu ao geólogo atender o telefone

‘The poet assured the geologist that he would answer the telephone’

d. O poeta forçou o geólogo a atender o telefone

‘The poet forced the geologist to answer the telephone’

21. a. A socióloga que socorreu a gestora gravou a reportagem

‘The sociologist that helped the manager recorded the news report’

b. A socióloga que a gestora socorreu gravou a reportagem

‘The sociologist that the manager helped recorded the news report’

c. A socióloga garantiu à gestora gravar a reportagem

‘The sociologist assured the manager that she would record the news report’

d. A socióloga forçou a gestora a gravar a reportagem

‘The sociologist forced the manager to record the news report’

22. a. A freira que perseguiu a porteira terminou o projeto

‘The nun that chased the portress finished the project’

b. A freira que a porteira perseguiu terminou o projeto

‘The nun that the portress chased finished the project’

c. A freira garantiu à porteira terminar o projeto

‘The nun assured the portress that she would finish the project’

d. A freira forçou a porteira a terminar o projeto

‘The nun forced the portress to finish the project’

23. a. O mágico que elogiou o alfaiate concluiu o curso

‘The magician that praised the tailor finished the course’

b. O mágico que o alfaiate elogiou concluiu o curso

‘The magician that the tailor praised finished the course’

c. O mágico garantiu ao alfaiate concluir o curso

‘The magician assured the tailor that he would finish the course’

d. O mágico forçou o alfaiate a concluir o curso

‘The magician forced the tailor to finish the course’

24. a. O arquiteto que enganou o economista assistiu à conferência

‘The architect that tricked the economist attended the conference’

b. O arquiteto que o economista enganou assistiu à conferência

‘The architect that the economist tricked attended the conference’

c. O arquiteto garantiu ao economista assistir à conferência

‘The architect assured the economist that he would attend the conference’

d. O arquiteto forçou o economista a assistir à conferência

‘The architect forced the economist to attend the conference’

25. a. O músico que criticou o pintor escreveu um livro

‘The musician that criticized the painter wrote a book’

b. O músico que o pintor criticou escreveu um livro

‘The musician that the painter criticized wrote a book’

c. O músico prometeu ao pintor escrever um livro

‘The musician promised the painter to write a book’

d. O músico convenceu o pintor a escrever um livro

‘The musician convinced the painter to write a book’

26. a. A fotógrafa que emocionou a detetive cumpriu o prazo

‘The photographer that thrilled the detective met the deadline’

b. A fotógrafa que a detetive emocionou cumpriu o prazo

‘The photographer that the detective thrilled met the deadline’

c. A fotógrafa prometeu à detetive cumprir o prazo

‘The photographer promised the detective to meet the deadline’

d. A fotógrafa convenceu a detetive a cumprir o prazo

‘The photographer convinced the detective to meet the deadline’

27. a. A banqueira que empurrou a cantora construiu um puzzle

‘The banker that pushed the singer built a jigsaw puzzle’

b. A banqueira que a cantora empurrou construiu um puzzle

‘The banker that the singer pushed built a jigsaw puzzle’

c. A banqueira prometeu à cantora construir um puzzle

‘The banker promised the singer to build a jigsaw puzzle’

d. A banqueira convenceu a cantora a construir um puzzle

‘The banker convinced the singer to build a jigsaw puzzle’

28. a. O professor que ignorou o taxista recebeu os convidados

‘The professor that ignored the taxi driver welcomed the guests’

b. O professor que o taxista ignorou recebeu os convidados

‘The professor that the taxi driver ignored welcomed the guests’

c. O professor prometeu ao taxista receber os convidados

‘The professor promised the taxi driver to welcome the guests’

d. O professor convenceu o taxista a receber os convidados

‘The professor convinced the taxi driver to welcome the guests’

29. a. A investigadora que acalmou a advogada preparou a festa

‘The researcher that calmed the lawyer prepared the party’

b. A investigadora que a advogada acalmou preparou a festa

‘The researcher that the lawyer calmed prepared the party’

c. A investigadora prometeu à advogada preparar a festa

‘The researcher promised the lawyer to prepare the party’

d. A investigadora convenceu a advogada a preparar a festa

‘The researcher convinced the lawyer to prepare the party’

30. a. O comediante que insultou o mecânico assumiu a culpa

‘The comedian that insulted the mechanic took the blame’

b. O comediante que o mecânico insultou assumiu a culpa

‘The comedian that the mechanic insulted took the blame’

c. O comediante prometeu ao mecânico assumir a culpa

‘The comedian promised the mechanic to take the blame’

d. O comediante convenceu o mecânico a assumir a culpa

‘The comedian convinced the mechanic to take the blame’

**Appendix II.** Results of the follow-up models.

Table 1. Results of the follow-up model assessing whether the relative clause type effect in accuracy was modulated by performance on supplementary tasks. “RC” = relative clause type (SR vs OR); “RSpan” = Reading Span; “BP-task” = Brown-Peterson task; “Vocabulary” = Vocabulary subtest (WAIS-III); “Fluency” = Semantic Fluency task.

| Variable | F | df1 | df2 | p |
| --- | --- | --- | --- | --- |
| RC | 46.43 | 1 | 1970 | <.001 |
| RSpan | 16.86 | 1 | 1970 | <.001 |
| BP-task | 1.14 | 1 | 1970 | .286 |
| Vocabulary | 1.48 | 1 | 1970 | .224 |
| Fluency | .03 | 1 | 1970 | .853 |
| RC*RSpan | .02 | 1 | 1970 | .884 |
| RC*BP-task | 1.06 | 1 | 1970 | .304 |
| RC*Vocabulary | .13 | 1 | 1970 | .723 |
| RC*Fluency | 1.52 | 1 | 1970 | .217 |

Table 2. Results of the follow-up model assessing whether the relative clause type effect in response times was modulated by performance on supplementary tasks. “RC” = relative clause type (SR vs OR); “RSpan” = Reading Span; “BP-task” = Brown-Peterson task; “Vocabulary” = Vocabulary subtest (WAIS-III); “Fluency” = Semantic Fluency task.

| Variable | F | df1 | df2 | p |
| --- | --- | --- | --- | --- |
| RC | 13.53 | 1 | 1645 | <.001 |
| RSpan | 1.50 | 1 | 1645 | .221 |
| BP-task | .01 | 1 | 1645 | .928 |
| Vocabulary | 3.51 | 1 | 1645 | .061 |
| Fluency | .42 | 1 | 1645 | .517 |
| RC*RSpan | 5.37 | 1 | 1645 | .021 |
| RC*BP-task | .00 | 1 | 1645 | .967 |
| RC*Vocabulary | 1.79 | 1 | 1645 | .181 |
| RC*Fluency | .07 | 1 | 1645 | .798 |

Table 3. Results of the follow-up model assessing whether the relative clause type effect in reading times at the post-critical region was modulated by performance on supplementary tasks. “RC” = relative clause type (SR vs OR); “RSpan” = Reading Span; “BP-task” = Brown-Peterson task; “Vocabulary” = Vocabulary subtest (WAIS-III); “Fluency” = Semantic Fluency task.

| Variable | F | df1 | df2 | p |
| --- | --- | --- | --- | --- |
| RC | 55.76 | 1 | 1632 | <.001 |
| RSpan | 4.46 | 1 | 1632 | .035 |
| BP-task | 5.35 | 1 | 1632 | .021 |
| Vocabulary | .41 | 1 | 1632 | .525 |
| Fluency | .05 | 1 | 1632 | .823 |
| RC*RSpan | .57 | 1 | 1632 | .449 |
| RC*BP-task | .40 | 1 | 1632 | .528 |
| RC*Vocabulary | .65 | 1 | 1632 | .420 |
| RC*Fluency | .45 | 1 | 1632 | .502 |

Table 4. Results of the follow-up model assessing whether the control type effect in response times was modulated by performance on supplementary tasks. “Control” = control type (SC vs OC); “RSpan” = Reading Span; “BP-task” = Brown-Peterson task; “Vocabulary” = Vocabulary subtest (WAIS-III); “Fluency” = Semantic Fluency task

| Variable | F | df1 | df2 | p |
| --- | --- | --- | --- | --- |
| Control | 7.07 | 1 | 1587 | .008 |
| RSpan | .79 | 1 | 1587 | .374 |
| BP-task | 1.18 | 1 | 1587 | .277 |
| Vocabulary | 1.12 | 1 | 1587 | .290 |
| Fluency | .01 | 1 | 1587 | .922 |
| Control*RSpan | .25 | 1 | 1587 | .614 |
| Control*BP-task | .58 | 1 | 1587 | .446 |
| Control*Vocabulary | .11 | 1 | 1587 | .736 |
| Control*Fluency | 1.47 | 1 | 1587 | .226 |

|  |
| --- |

Table 5. Results of the follow-up model assessing whether the control type effect in reading times at the critical region was modulated by performance on supplementary tasks. “Control” = control type (SC vs OC); “RSpan” = Reading Span; “BP-task” = Brown-Peterson task; “Vocabulary” = Vocabulary subtest (WAIS-III); “Fluency” = Semantic Fluency task

| Variable | F | df1 | df2 | p |
| --- | --- | --- | --- | --- |
| Control | 61.93 | 1 | 1566 | <.001 |
| RSpan | 1.90 | 1 | 1566 | .169 |
| BP-task | 9.48 | 1 | 1566 | .002 |
| Vocabulary | 1.22 | 1 | 1566 | .271 |
| Fluency | 1.06 | 1 | 1566 | .303 |
| Control*RSpan | 1.04 | 1 | 1566 | .308 |
| Control*BP-task | .90 | 1 | 1566 | .344 |
| Control*Vocabulary | .10 | 1 | 1566 | .755 |
| Control*Fluency | .02 | 1 | 1566 | .902 |
